# Phylogenomics and Biogeography of the Mammilloid Clade Revealed an Intricate Evolutionary History Arose in the Mexican Plateau

**DOI:** 10.3390/biology12040512

**Published:** 2023-03-29

**Authors:** Delil A. Chincoya, Salvador Arias, Felipe Vaca-Paniagua, Patricia Dávila, Sofía Solórzano

**Affiliations:** 1 Laboratorio de Ecología Molecular y Evolución, UBIPRO, FES Iztacala, Universidad Nacional Autónoma de México, Avenida de los Barrios 1, Los Reyes Iztacala, Tlalnepantla de Baz 54090, Estado de México, Mexico; 2 Posgrado en Ciencias Biológicas, Unidad de Posgrado, Edificio D, 1° Piso, Circuito de Posgrados, Ciudad Universitaria, Coyoacán 04510, Ciudad de México, Mexico; 3 Jardín Botánico, Instituto de Biología, Universidad Nacional Autónoma de México, Tercer Circuito Exterior, Ciudad Universitaria, Coyoacán 04510, Ciudad de México, Mexico; 4 Laboratorio Nacional en Salud, Diagnóstico Molecular y Efecto Ambiental en Enfermedades Crónico-Degenerativas, FES Iztacala, Universidad Nacional Autónoma de México, Los Reyes Iztacala, Tlalnepantla de Baz 54090, Estado de México, Mexico; 5 Subdirección de Investigación Básica, Instituto Nacional de Cancerología, Tlalpan 14080, Ciudad de México, Mexico; 6 Unidad de Investigación en Biomedicina, FES Iztacala, Universidad Nacional Autónoma de México, Los Reyes Iztacala, Tlalnepantla de Baz 54090, Estado de México, Mexico; 7 Laboratorio de Recursos Naturales, UBIPRO, FES Iztacala, Universidad Nacional Autónoma de México, Avenida de los Barrios 1, Los Reyes Iztacala, Tlalnepantla de Baz 54090, Estado de México, Mexico

**Keywords:** arid lands, biogeography, Cactaceae, colonization, *Mammillaria*, Mexican Plateau, Miocene, phylogenomics, Pleistocene, recent diversification

## Abstract

**Simple Summary:**

Cacti account for nearly 1440 species, most of them native to the American continent. These succulent plants are the most ubiquitous elements of the arid ecosystems. Mexico harbors the highest number of cacti species in the world (45%). Unfortunately, many of them are threatened by human activities. Although having this biodiversity relevance, presently the evolutionary processes of cacti have been poorly studied. Because the biological and conservation unit is the species, evolutionary studies provide relevant information. In this study, we analyzed how and when past events shaped the evolutionary relationships of 103 species. Our results showed that from 4.5 million years ago the arid regions of Mexico were the locations for abundant cacti speciation. From these lands, cacti have colonized most of the Mexican territories, the southern regions of the United States, as well as the Caribbean. The evolution of these plants was probably promoted by past temperatures that were comparable to the present ones. We identified different speciation and dispersal events in these fascinating plants. This study identified the Mexican Plateau as the place where the early stages of the evolutionary history of cacti occurred.

**Abstract:**

Mexico harbors ~45% of world’s cacti species richness. Their biogeography and phylogenomics were integrated to elucidate the evolutionary history of the genera *Coryphantha*, *Escobaria*, *Mammillaria*, *Mammilloydia*, *Neolloydia*, *Ortegocactus*, and *Pelecyphora* (Mammilloid Clade). We analyzed 52 orthologous loci from 142 complete genomes of chloroplast (103 taxa) to generate a cladogram and a chronogram; in the latter, the ancestral distribution was reconstructed with the Dispersal-Extinction-Cladogenesis model. The ancestor of these genera arose ~7 Mya on the Mexican Plateau, from which nine evolutionary lineages evolved. This region was the site of 52% of all the biogeographical processes. The lineages 2, 3 and 6 were responsible for the colonization of the arid southern territories. In the last 4 Mya, the Baja California Peninsula has been a region of prolific evolution, particularly for lineages 8 and 9. Dispersal was the most frequent process and vicariance had relevance in the isolation of cacti distributed in the south of Mexico. The 70 taxa sampled as *Mammillaria* were distributed in six distinct lineages; one of these presumably corresponded to this genus, which likely had its center of origin in the southern part of the Mexican Plateau. We recommend detailed studies to further determine the taxonomic circumscription of the seven genera.

## 1. Introduction

The integration of the analytical frameworks of phylogenetics and biogeography allows analysis of the influence of biogeography on the evolutionary history of extant taxa, as well as to identify all those biogeographical events that promoted speciation [1]. The studies that incorporate these frameworks have inferred past biogeographical scenarios that have shaped the current geographical ranges of the species (e.g., [2,3]) and even large flora assemblies (e.g., [4,5,6]). Furthermore, they have enabled evaluation of the relative role of vicariance and dispersal in shaping the current geographical distribution of species [1,7,8]. Gradually, with the advances of high throughput sequencing technologies, more of these studies are using denser molecular sampling, which has made it possible to obtain confident phylogenetic trees that may serve to resolve close phylogenetic relationships [9]. Poor molecular sampling usually produces non-monophyletic trees, or discordance with phylogenies based on morphology [10]. In addition, the biogeographical data may support the establishment of taxonomic limits between species [10], and actually they have been identified as providing better auxiliary information than morphology to elucidate phylogenetic relationships [11].

In addition, it is recognized that global paleoclimatic changes have shaped the large current distribution patterns of the biota and caused extinctions at different geographical scales [12]. Furthermore, they influenced the expansion and contraction of the geographical distribution of the current extant species (e.g., [13,14]). Consequently, paleoclimate changes have been recognized as one of the most influential factors in shaping the world biodiversity patterns at large scales, but also for understanding the current local flora assemblies (e.g., [15]). On the other hand, the topography and the intricate local orography have also influenced the ecological, biogeographical, and evolutionary processes of the local biota [16]. All these events and processes that occurred in the past might have modified gene flow patterns, which gradually may cause population genetic divergence and eventually promoted speciation processes [17].

In the contemporary arid lands of the American continent, many complex assemblages of native local floras are found in which cacti taxa are the most ubiquitous elements. The nearly 1440 taxa grouped in Cactaceae [18] are recognized as a monophyletic group [19]. Today the evolutionary history of Cactaceae, particularly its origin and mode of speciation, are still considered enigmatic [20]. Due to the lack of fossil records of Cactaceae representatives, there is no direct evidence to date its origin. However, estimations based on molecular clock hypothesis have dated the origin of Cactaceae to nearly 28.8 million years ago (Mya) [21], or 32.11 Mya [22], and 35 Mya [23]. Accordingly, these estimations place the origin of Cactaceae in the Cenozoic Era, in the Paleogene period from the Late Eocene (~35 Mya) to the Middle Oligocene (~28 Mya). In addition, Arakaki et al. [23] concluded that unequal and inconstant speciation rates for 123 cacti sampled were explained by the environmental changes that occurred in the Miocene, based on the phylogenetic tree obtained with two loci, one from the nuclear genome (PHYC) and the other one from the chloroplast (trnK/matK). Accordingly, these authors suggested that there have been at least six main peaks of speciation in the evolutionary history of Cactaceae. These authors dated the earliest two speciation peaks to 25 Mya and 15 Mya, whereas the other four occurred in the last 8 million years. Furthermore, they showed that those last four peaks were contemporaneous to the decreases in atmospheric CO_2_ that promoted global aridification, giving new ecological opportunities to cacti [23]. On the other hand, specialized paleoclimatic studies (e.g., [24,25]) have dated the decreases of atmospheric CO_2_ from the Middle Miocene (14 Mya) to the Middle Pleistocene (0.8 Mya). These relatively low levels of CO_2_ eventually caused a cooler and drier global climate, a phenomenon recognized as an aridification process [26].

In Cactaceae, the genus *Mammillaria* Haw is notable for its diversity [27], conservation concerns [28] and unresolved phylogenetic and taxonomic issues [18]. The taxonomy of *Mammillaria* has been controversial from its original description. In 1753, Charles Linnaeus described the type specimen as *Cactus mammillaris* L. and later it was renamed as *Mammillaria* in 1812 [29]. During its history, the genus *Mammillaria* has received 14 different names [27], reflecting the difficulty in achieving a clear taxonomic circumscription based almost entirely in external morphological traits. Throughout the last two centuries, numerous attempts have been made to organize the wide infrageneric morphological variation among taxa classified as *Mammillaria* (e.g., [30,31,32]. The most recent infrageneric classification was proposed by Hunt [18], who recognized eight subgenera and 15 series. The subgenus *Mammillaria* contains the highest number of species (117), followed by *Chilita* Orcutt (18) and *Krainzia* Backeb. (12); whereas *Cochemiea* Brandegee, *Dolichothele* (K.Schum.) Britton & Rose, *Mammillopsis* Morren, *Oehmea* Buxb., and *Phellosperma* Britton & Rose together add 16 species. For the purposes of the present study, we follow this last infrageneric classification system; nevertheless, phylogenetic support for these infrageneric classifications has not been tested.

Today, the global geographical distribution of *Mammillaria* ranges from the southern arid lands of the United States to the north of South America. Mexico has the highest documented diversity of *Mammillaria*. Nearly 20% of the species of *Mammillaria* are distributed in the Mexican arid lands of the southern part of the Chihuahuan Desert [33]. Only two of the 163 species currently recognized [18], *M. mammillaris* (L.) H. Karst and *M. nivosa* Link ex Pfeiff. are not documented in this country [33]. The genus *Mammillaria* is a rare taxon across Central America, as only four species are recorded in Guatemala (*M. albilanata* Backeb., *M. columbiana* Salm-Dyck, *M. ericantha* Link & Otto ex Pfeiff. and *M. voburnensis* Scheer), and two of them are also distributed across Nicaragua and Honduras (*M. columbiana* and *M. voburnensis*). Two more, *M. columbiana* and *M. mammillaris*, are documented in some small localities in the north of Venezuela and Colombia. In addition, four species (*M. columbiana*, *M. mammillaris*, *M. nivosa*, and *M. prolifera* (Mill.) Haw.) are recorded in the Caribbean islands [33].

The early phylogenetic studies carried out with *Mammillaria* reignited the unsolved discussion regarding its unclear taxonomic circumscription and its limits with taxa of another six genera (*Coryphantha* (Engelm.) Lem., *Escobaria* Britton & Rose, *Mammilloydia* Buxb., *Neolloydia* Britton & Rose, *Ortegocactus* Alexander, and *Pelecyphora* Ehrenb.). These six genera and *Mammillaria* compose the Mammilloid Clade [34]. Butterworth and Wallace [35] analyzed the phylogenetic relationships of 123 species of Mammilloid Clade (113 of them grouped in *Mammillaria)* based on two plastid loci (*rpl16* intron and the intergenic spacer *psbA-trnH*). Their phylogenetic tree showed abundant polytomies and low support bootstrap values. In addition, the sampled taxa of these six genera were grouped together with those of *Mammillaria*. Hence the authors concluded that this genus has a polyphyletic origin. Later, Crozier [36] used 10 plastid loci to analyze 157 cacti taxa; only 29 of them were *Mammillaria* taxa and 10 belonged to the six genera. The results of this study did not resolve the phylogenetic relationships of the sampled taxa; it also concluded non-monophyly for *Mammillaria*. In addition, Crozier [36] concluded that the monophyly of *Mammillaria* could only be obtained if: (1) the *Mammillaria* genus was expanded to include all the species currently grouped in the six genera; or (2) the genus *Mammillaria* includes only those species of the subgenus *Mammillaria* sensu Hunt [37]. Breslin et al. [38] recently sampled 93,808 bp of the large single copy (LSC) of the chloroplast genome from 78 cacti taxa, 52 of which were *Mammillaria* and 17 from five genera (*Coryphantha*, *Escobaria*, *Neolloydia*, *Ortegocactus*, and *Pelecyphora*). These authors concluded monophyly for *Mammillaria* by excluding all those species that were grouped in a distinct clade, which was composed of taxa in *Mammillaria*, *Neolloydia*, and *Ortegocactus*. In addition, it was proposed that all the species of this clade to be placed inside the genus *Cochemiea.*

In this study, we integrated phylogenomics and historical biogeography to elucidate the controversial evolutionary history of the group of seven genera of cacti (*Coryphantha*, *Escobaria*, *Mammillaria*, *Mammilloydia*, *Neolloydia*, *Ortegocactus*, and *Pelecyphora*) sensu Hunt [18]. We hypothesized that these taxa have a monophyletic origin, whose unique ancestor arose recently and rapidly evolved in response to past decreases in global temperature. The objectives were to evaluate the phylogenetic relationships of the studied species, to estimate their divergence times, and to identify the probable ancestral geographical distribution of the taxa studied in these seven genera; to discuss the possible effects of past global temperature and orographic events in the colonization and expansion of these cacti across the arid lands of Mexico; and finally we use our results to identify the taxonomic limits of the genera studied with emphasis on the taxa sampled in the genus *Mammillaria*.

## 2. Materials and Methods

### 2.1. Taxon Sampling

A total of 142 complete chloroplast genomes (cpDNA) of 103 taxa were analyzed (Table S1), of which 141 cpDNA belong to the tribe Cacteae (Cactoideae). The non Cactoideae taxon *Blossfeldia liliputana* Werderm. was included because it was identified as the sister species for the rest of the subfamily Cactoideae [36]. We compiled these cpDNA from the following sources: seven complete cpDNA of *Mammillaria* previously published [39], as well as the raw data of 86 genomes that were downloaded from NCBI site, which were linked to BioProject PRJNA671701 [38]. In addition, the whole complete chloroplast genomes of 49 taxa were de novo sequenced in this study. The tissue samples for 47 of these taxa were provided by the collection of the Botanical Garden of the Universidad Nacional Autónoma de México, whilst the tissues of *M. napina* J.A.Purpus and *M. huitzilopochtli* D.R.Hunt were obtained from completed research projects (SS). Among these 142 genomes, 132 represented seven of the genera (i.e., Mammilloid Clade): *Coryphantha*, *Escobaria*, *Mammillaria*, *Mammilloydia*, *Neolloydia*, *Ortegocactus*, and *Pelecyphora* (Table 1).

**Figure 1:**
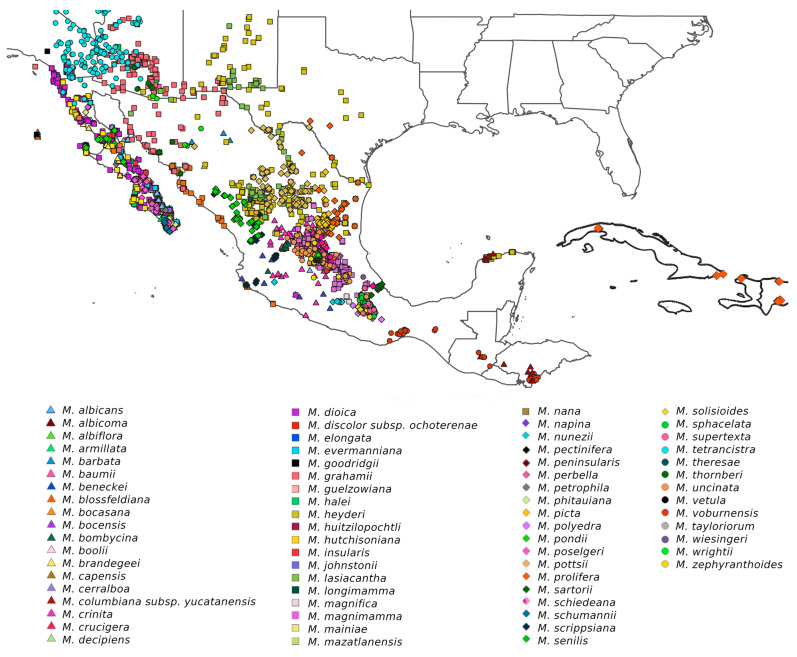
World geographical distribution of the 70 taxa of *Mammillaria*. Geographical distribution per taxon is showed in detail in Supplementary Materials (File S1).

In addition, the taxonomic sampling covered the whole geographical range of five of these genera (*Coryphantha*, *Mammilloydia*, *Neolloydia*, *Ortegocactus*, and *Pelecyphora*). In contrast, the geographical range of *Mammillaria* was not sampled in South America; and for *Escobaria* was not sampled the Caribbean. Of them, 105 specimens (70 taxa) corresponded to *Mammillaria*, which are currently distributed in continental and peninsular Mexican territories, as well as the southern parts of the USA and the Caribbean. We documented the geographical distribution in the Global Biodiversity Information Facility (GBIF) ((https://www.gbif.org/ (accessed on 20 March 2022)) (Figure 1). In order to reduce record density, we used the spThin package [40] to discard all those records with <1 km in separation distance. The geographical data of those remained records were hand-curated following to Hernández and Gómez-Hinostrosa [33]. In addition, we sampled as external groups 10 specimens of eight genera: *Acharagma* (N.P. Taylor) Glass, *Ariocarpus* Scheidw., *Blossfeldia* Werderm., *Cumarinia* (Knuth) Buxb., *Lophophora* J.M. Coult., *Stenocactus* (K. Schum.) A. Berger, *Strombocactus* Britton & Rose, and *Turbinicarpus* Backeb.

### 2.2. DNA Extraction, cpDNA Enrichment, and High-Throughput Sequencing

For each of the 49 species de novo sequenced, 30–100 mg of frozen tissue was obtained to isolate 1 ug of gDNA with A260/280 ratio ≥ 1.7. The tissue samples were individually processed with the DNeasy plant mini kit (Qiagen, Hilden, Germany), following the manufacturer’s instructions. To obtain an enriched proportion of chloroplast genome, these gDNAs were processed with the NEBNext Microbiome DNA Enrichment Kit (New England BioLabs, Ipswich, MA, USA) according to the kit’s instructions. These enriched DNAs were used to prepare pair-end (PE) genomic libraries with the Nextera XT kit, with mean insert size of 400 bp, and were sequenced in MiSeq 2 × 300 cycles.

### 2.3. De Novo Assembly of Chloroplast Genomes

We assembled de novo the raw data of the 86 genomes attached to Breslin et al. [38] (Table S1); as well as the raw data of the 49 taxa de novo sequenced. These 135 genomes were filtered, trimmed and adapters were removed with TrimGalore version 0.4.3 [41]. The recovered reads with PHRED quality score ≥ 15 and length ≥ 80 bp were assembled with Get Organelle version v1.7.1 [42], using as a seed the cpDNA of *M. supertexta* (GenBank accession: MN508963.1) previously published [39].

### 2.4. Phylogenetic Relationships and Divergence Times

The 142 genomes (Table 1) were analyzed with BLAST version 2.5.0 [43] to identify common loci based on sequence similarity. All these common loci were aligned with MAFFT version v7.310 [44]. Because the genomes analyzed showed different structural arrangements, some sequences were not recovered, giving alignments with a high proportion of missing data; and other alignments showed low molecular variation. Thus, these two types of alignments were discarded before further phylogenetic analysis. Accordingly, only those alignments with sequences present for ≥70% of the studied taxa, ≥15% of proportion of informative sites (PIS) and a length of ≥200 bp were obtained. Accordingly, a total of 52 orthologous loci (Table S2) were identified and concatenated in a matrix of 48,869 bp used for the phylogenomic analysis and estimation of times of divergence. The matrix partitions and substitution models were estimated with ModelFinder [45], implemented in IQ-TREE2 version 2.1.4-beta [46]. The phylogenetic tree was generated with IQ-TREE2 using *B. liliputana* as the outgroup and running 10,000 ultra-fast bootstrap (UFBoot) replicates. Then, we estimated the evolutionary times of divergences using two secondary calibrations from previous estimations for the Cactaceae family [22]. In our first calibration, we used the crown age of 12.67–24.46 Mya estimated for the whole Cactoideae subfamily, and for the second calibration, we used the crown age of 4.86–10.63 Mya for the clade composed of the seven focus genera (*Coryphantha*, *Escobaria*, *Neolloydia*, *Mammillaria*, *Mammilloydia*, *Ortegocactus*, and *Pelecyphora*). We estimated the divergence times with BEAST version v2.6.6 [47], whose specific input file was constructed with BEAUti. In this input file was specified the GTR + I + Γ substitution model, which was estimated with Modeltest [48] according to AICc, a lognormal relaxed molecular clock, calibration points as uniform distributions, a Yule process tree prior, and 200,000,000 generations with a sampling frequency of each 2000 generations. In addition, convergence of parameter estimation was corroborated with Tracer version v1.7.2 [49], and the trees were summarized in a maximum clade credibility tree with TreeAnnotator version v2.6.3 [50]; 10% of the trees were discarded based on this final analysis.

### 2.5. Biogeographical Analysis

For the biogeographical analysis we documented the current geographical distribution for each of the 141 specimens (102 taxa) native to the arid lands of North America. As *B. liliputana* is endemic to South America it was discarded from the biogeographical analysis. The geographical data were compiled from GBIF ((https://www.gbif.org/ (accessed on 20 March 2022)). These data were verified by checking the geographical distribution of taxa reported from different sources [33,51,52]. The geographical distribution range of the 141 specimens was classified into the respective Mexican Floristic Provinces proposed by Rzedowski [53]. We estimated the ancestral geographical ranges based on the dated tree using the R package BioGeoBEARS version 1.1.1 [54] implemented in RASP4 v4.0 [55]. We evaluated four distinct models of the geographical range evolution for the 141 specimens: both the model of Dispersal-Extinction-Cladogenesis (DEC); and the likelihood version of Dispersal-Vicariance Analysis (DIVALIKE) were tested under two conditions, with and without the assumption of Founder-Event Speciation (+J) parameter. Finally, we plotted the changes estimations in the global surface air temperature (ΔT) in relation to the current values, previously published in the supplementary material (S4) of Herbert et al. [26].

## 3. Results

### 3.1. Evolutionary History of Cacti: Recent Divergence and Intricate Biogeography

**Figure 2:**
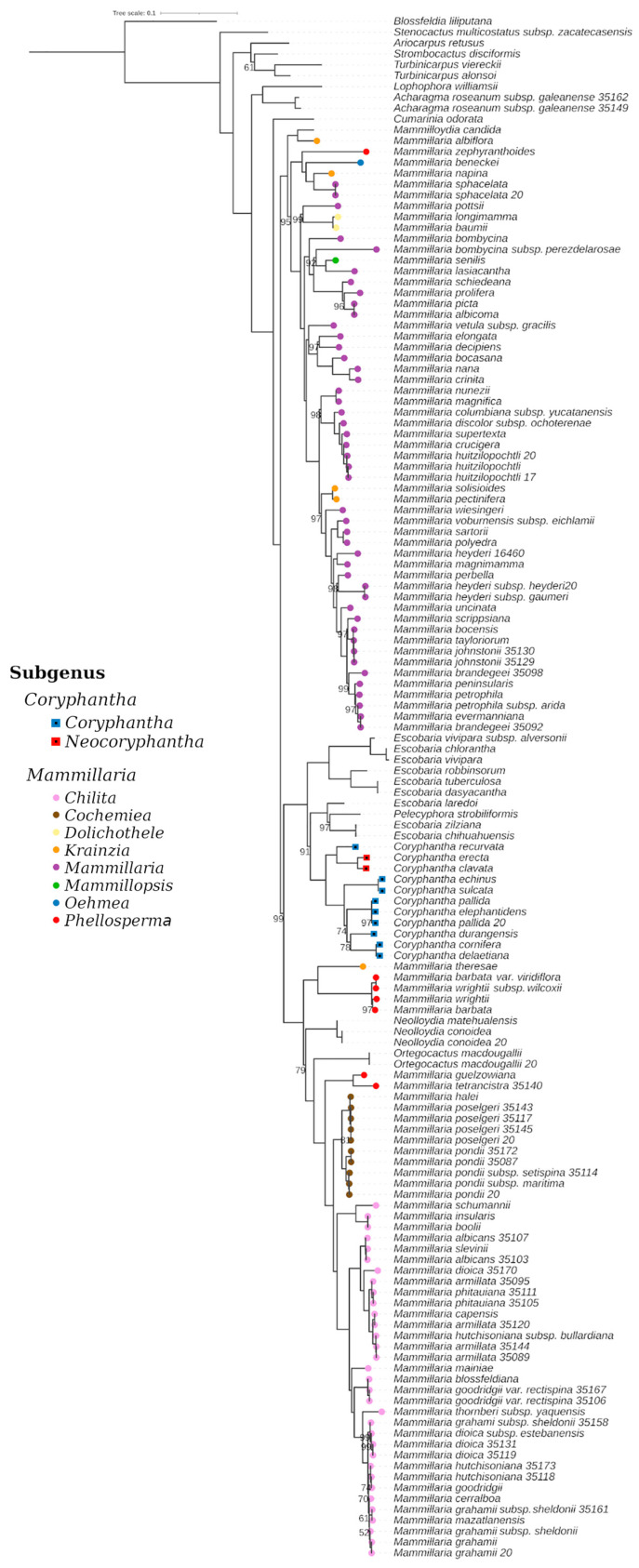
Phylogenetic tree estimated with IQ-TREE2 for the 103 taxa (142 specimens) using *B. liliputana* as the outgroup. Numbers below the nodes indicate UFBoot values < 100. Colored circles and squares indicate subgenera for *Mammillaria* and *Coryphantha*, respectively.

**Figure 3:**
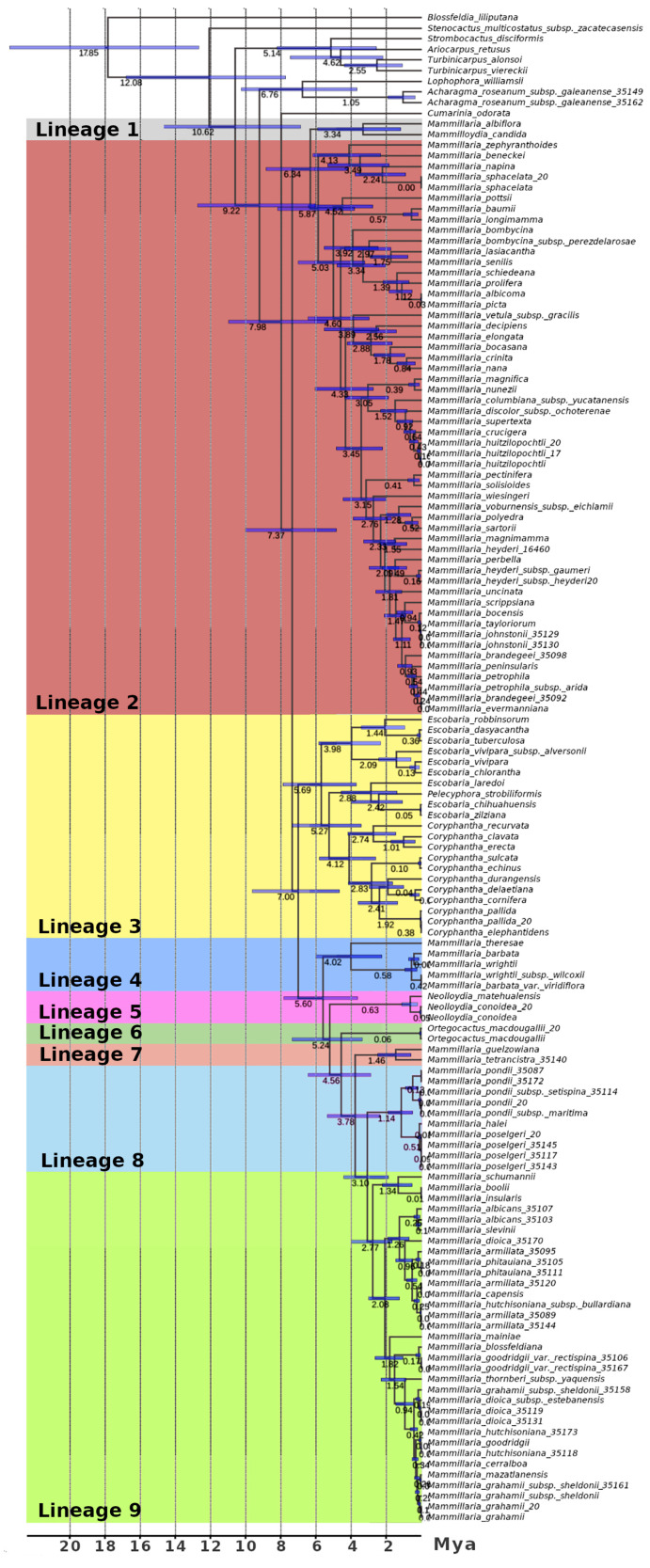
Chronogram estimated for the 103 taxa (142 specimens). The maximum clade credibility tree shows the divergence times estimated in BEAST. Blue bars represent 95% HPD intervals for the node ages. Shadow colors show the nine evolutionary lineages identified.

**Figure 4:**
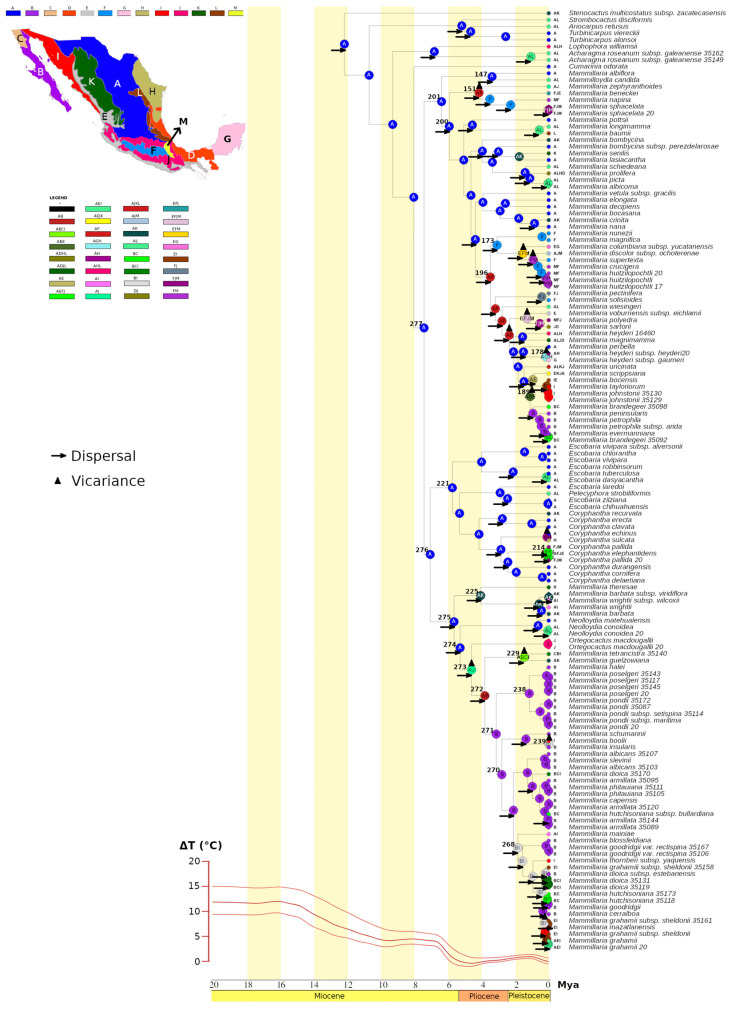
Ancestral geographical distribution of the North American taxa estimated on the chronogram. The estimation of the most likely ancestral distribution is represented in a colored circle for each node of the tree. The letters in the map and in the tree corresponded to Baja California (B), Balsas Basin (F), California (C), Gulf of Mexico Coast (D), Mexican Plateau (A), Meridional Serranias (J), Northeast Coastal Plain (H), Northwest Coastal Plain (I), Pacific Coast (E), Sierra Madre Occidental (K), Sierra Madre Oriental (L), Tehuacan Valley (M), and Yucatan Peninsula (G). The nodes of the main evolutionary events were numbered (see the text). The estimated events of dispersal and vicariance are indicated by arrows and triangles, respectively. The letters besides the tips indicate the current geographical distribution of the taxa. At the bottom of the figure were drawn the changes estimations in the global surface air temperature (ΔT).

The topologies of the phylogenetic tree (ML tree) (Figure 2) and the chronogram (BI tree) (Figure 3) were highly concordant; the only difference was found in the relationships of the small clade composed of *Ariocarpus*, *Strombocactus*, and *Turbinicarpus*. In the ML tree, the clade *Turbinicarpus*-*Strombocactus* was sister to *Ariocarpus*, whereas in the BI tree, the *Turbinicarpus*-*Ariocarpus* clade was sister to *Strombocactus*. In the biogeographical analysis, the DEC model (without +J parameter) was selected according to the value of AICcWt (File S2), however, this value was slightly higher than that obtained for the DEC+J model. In addition, these two models provided very similar estimations of the ancestral geographical distribution (Figures S1 and S2). The biogeographical analysis estimated a total of 135 dispersal events and 13 vicariant events (Figure 4).

The phylogenetic results (Figure 2) clearly identified for the 102 taxa (141 specimens) of the Cacteae tribe a common ancestor, which arose in the Mexican Plateau in the Late Miocene ~12.08 Mya (95% HPD: 7.73–16.82) (Figure 3). According to the temperatures taken from the bibliography [26], between 15 and 9 Mya there was a drastic decrease in global temperature of ΔT~8 °C. In this period, our results showed two key phylogenetic splits in Cacteae: the first one that separated *Stenocactus* from the remaining 101 taxa; followed by the second one that separated *Ariocarpus*, *Strombocactus* and *Turbinicarpus* from the remaining 96 taxa (Figure 4). Later, during a short period of nearly 2.7 million years (from 9 to 6.3 Mya), the temperature stayed stable (ΔT~0.1), and during this period two splits occurred. The first one is represented by the separation of *Acharagma* and *Lophophora*; the second one consists of the separation of the ancestor of *Cumarinia.* In this period (9 to 6.3 Mya), we identified the beginning of the complex evolutionary history of the 93 taxa belonging to the Mammilloid Clade (Figure 4). Moreover, these taxa continued their diversification processes during the next period of two million years (between 6.3 and 4.3 Mya), when the temperature again declined (ΔT~4.4 °C). These diversification processes continued and intensified during the last 4.3 Mya. In this last 4.3 million years, the temperature has not been steady; from 4.3 to 1 Mya (Late Pliocene to Pleistocene) a slight increase in temperature (ΔT~1 °C) was documented. However, in the last 1 million years, the temperature has decreased (ΔT~0.5 °C) (Figure 4).

The chronogram showed that the ancestor of the Mammilloid Clade originated nearly 7.37 Mya (95% HPD: 4.86–10.02 Mya). This early ancestor (node 277, Figure 4) arose at the end of the Miocene when the value ΔT of the temperature was low (ΔT~0.1 °C). This ancestor had as its probable ancestral geographical area the Mexican Plateau (Figure 4). Eventually, from this ancestor nine independent evolutionary lineages were derived (Figure 3); which profusely diversified in the last 4.3 Mya, when little increase-decrease of temperature occurred (Figure 4). This early common ancestor diverged into two new ancestors, one of which (node 276, Figure 4) was dated nearly 7 Mya (95% HPD: 4.67–9.64 Mya) (Figure 3). This ancestor had as its ancestral biogeographical scenario the Mexican Plateau (Figure 4), and from it evolved those taxa currently grouped in the six genera (*Coryphantha*, *Escobaria*, *Mammillaria*, *Neolloydia*, *Ortegocactus*, and *Pelecyphora*). The other ancestor (node 201, Figure 4) arose 6.34 Mya (95% HPD: 4.17–8.86 Mya) (Figure 3), probably also in the Mexican Plateau. From this lat ancestor evolved those taxa that were grouped in two genera: *Mammillaria* and *Mammilloydia*.

A conspicuous result obtained was that those 70 taxa (105 specimens) sampled as *Mammillaria* were distributed in two main independent clades (nodes 201 and 275, Figure 4). The immediate ancestors of these two clades originated in the past Mexican Plateau. However, those taxa derived from them clearly differ in their evolutionary history (Figure 4). Accordingly, the taxa sampled as genus Mammillaria, in fact, were distributed in six different and independent evolutionary lineages, each one with its own evolutionary history (Figure 3 and Figure 4).

### 3.2. Evolutionary History of the Nine Lineages

#### 3.2.1. Evolutionary Lineage 1

The short evolutionary lineage 1 was composed only of *Mammilloydia candida* (Scheidw.) Buxb. and *Mammillaria albiflora* Backeb, whose immediate ancestor was dated to nearly 3.34 Mya (95% HPD: 1.19–5.92 Mya). This ancestor (node 147, Figure 4) probably arose on the Mexican Plateau, during the Middle Pliocene, when the temperature underwent a slight increase (ΔT~1 °C) (Figure 4). At the present time, *M. candida* and *M. albiflora* are distributed in a small region on the southern region of the Mexican Plateau, and *M. candida* extends its geographical range to the northwest of this biogeographical area (Figure 4). Additionally, lineage 1 was identified as the phylogenetic sister to lineage 2 (Figure 2).

#### 3.2.2. Evolutionary Lineage 2

The most probable ancestral geographical area for the immediate ancestor of lineage 2 was the Mexican Plateau (node 200, Figure 4). Lineage 2 arose nearly 5.87 Mya (95% HPD: 3.82–8.2), at the end of the Late Miocene, when the temperature decreased (ΔT~4.4 °C). However, most of the divergent processes in this lineage occurred in the last 4.3 million years, when a slight increase in the temperature (ΔT~1 °C) was followed by a slight decrease (ΔT~0.5 °C). This lineage grouped 45 of the sampled taxa, of which 37 taxa (82%) correspond to the subgenus *Mammillaria*, whereas the other eight taxa belong to five different subgenera (Figure 2). Three of these taxa (*M. napina*, *M. pectinifera* F.A.C. Weber, and *M. solisioides* Backeb.) corresponded to the subgenus *Krainzia*; two (*M. baumii* Boed and *M. longimamma* DC.) to the subgenus *Dolichothele*; one (*M. senilis* Lodd. ex Salm-Dyck) to *Mammilliopsis*; one (*M. beneckei* Ehrenb.) to the subgenus *Oehmea*; and finally, one species (*M. zephyranthoides* Scheidw.) to *Phellosperma*. Currently, 21 of these 45 taxa are endemic only to one of the 13 biogeographical areas (right-side letters beside the taxa in Figure 4); with the Mexican Plateau the area that has the highest number of endemics (eight taxa). In addition, most of the divergent events occurred in three biogeographical areas, which was the unique ancestral area or in conjunction with other ones: the Mexican Plateau (A) involved 66 % of the divergence events, the Balsas Basin (F) 35%, and the Tehuacan Valley (M) 13%.

Furthermore, biogeographical results suggested that such divergence processes were closely associated to the taxa dispersal towards new areas inside and outside of the Mexican Plateau (Figure 4). Accordingly, in the lineage 2 the long-distance dispersal has been a common phenomenon during the last ~4 million years (Figure 4). During these long-distance dispersal events, it seems that the ancestors moved out of the Mexican Plateau and eventually displaced along different routes, either via continental arid lands or crossing the sea (Gulf of Mexico and Gulf of California). During the Pliocene, we identified two independent events of colonization (nodes 151 and 196; Figure 4) to the arid southern Mexican territories (F, J and M; Figure 4), where the colonizers eventually speciated in situ (nodes 151 and 173; Figure 4). The first long-dispersal event occurred 4.13 Mya, and the second one was dated 3.45 Mya. These two events occurred during a slight increase of temperature (ΔT~1 °C) (Figure 4). These two colonization events took place from the Mexican Plateau (A) to the Balsas Basin (F), and from there to the adjacent areas of Tehuacan Valley (M) and Meridional Serranias (J) (Figure 4). In addition, we identified that during the Pleistocene, another two independent colonization events occurred towards the northern Mexican territories. The results indicate (Figure 4) that from the Mexican Plateau there was another dispersal route that took place along the foothills of Sierra Madre Occidental (A, K), crossing it, and reaching the Pacific slope of this Sierra. In addition, we identified a recent dispersal event dated nearly 1.11 Mya (node 189, Figure 4), in which an ancestor undertook a vicariant event, which separated two lineages, one of which diversified in the Baja California Peninsula (B) and the other in the northwest of continental Mexico (A, E) (Figure 4). On the other hand, on the eastern side of Mexico, another independent long-distance dispersal event was identified (node 178, Figure 4), crossing the Gulf of Mexico and reaching the Yucatán Peninsula nearly 0.16 Mya.

#### 3.2.3. Evolutionary Lineage 3

We estimated the origin of the ancestor of this lineage (node 221, Figure 4) was in the Mexican Plateau (A) at 5.69 Mya (95% HPD: 3.72–7.89 Mya) (Figure 3). This lineage grouped 19 of the sampled taxa belonging to the genera *Coryphantha* (10), *Escobaria* (8), and *Pelecyphora* (1). Currently, 17 of these 19 taxa are distributed in the northern region of the Mexican Plateau (Figure 4), and only two taxa of *Coryphantha* are distributed in the southern arid lands (F, J and M, Figure 4), suggesting that a long-distance dispersal event allowed *Coryphantha* to reach the southern arid lands of Mexico (Figure 4). Therefore, in this lineage most of the past divergent processes were identified as on the Mexican Plateau, and eventually moving to northern and southern Mexico (Figure 4). The majority of these processes were dated to the Late Pliocene, when there was a slight temperature increase (ΔT~1 °C) (Figure 4). Clearly, the phylogenetic relationships of this clade were fully resolved. These results recovered *Coryphantha* as monophyletic, whereas *Escobaria* is paraphyletic with respect to *Coryphantha* and *P*. *strobiliformis* (Figure 2).

These findings showed that the lineage 3 was the phylogenetic sister of a clade comprising six lineages (lineages 4–9) (Figure 3) that evolved from a common ancestor (node 275, Figure 4). This ancestor was dated to nearly 5.60 Mya (95% HPD: 3.62–7.84; Figure 3) and arose in the ancestral arid lands of the Mexican Plateau (Figure 4).

#### 3.2.4. Evolutionary Lineage 4

The lineage 4 was derived from an ancestor (node 225, Figure 4) dated nearly 4.02 Mya (95% HPD: 2.27–6.01 Mya), which had its ancestral geographical area on the Mexican Plateau (A) and the foothills of the Sierra Madre Occidental (K). We identified an early split that separated subgenus *Krainzia* (*M. theresae* Cutak, Figure 2) from *Phellosperma* (*M. barbata* Engelm. and *M. wrightii* Engelm.). In this last subgenus, a recent divergence (0.58 Mya, Figure 3) was identified; presently the taxa of this lineage are distributed in the northwestern territories of the Mexican Plateau, the Sierra Madre Occidental and the Northwest Coastal Plain (A, K and I, Figure 4).

#### 3.2.5. Evolutionary Lineage 5

Our results revealed a common ancestor (node 274, Figure 4) that originated lineage 5 and the other four independent lineages identified as lineages 6–9 (Figure 3). This ancestor was dated to 5.24 Mya (95% HPD: 3.38–7.38 Mya) and probably arose in the ancestral lands of the Mexican Plateau (Figure 4). Although the phylogenetic split of the ancestor (node 274, Figure 4) was dated to nearly 5 Mya, the origin of lineage 5 is very recent, as it was dated to 0.63 Mya (95% HPD: 0.23–1.13 Mya) (Figure 3), when a slight temperature decrease (ΔT~0.5 °C) occurred (Figure 4). The ancestral geographical area of this lineage was also the Mexican Plateau (A, Figure 4). In addition, this lineage currently groups the two taxa recognized in the genus *Neolloydia*, which are distributed in the Mexican Plateau (Figure 4). However, *N. matehualensis* Backeb. is endemic to the center of the Mexican Plateau, and *N. conoidea* (DC) Britton & Rose ranges from the southern to the northern range of the Mexican Plateau and reaches the southern arid lands of the USA.

#### 3.2.6. Evolutionary Lineage 6

Lineage 6 was composed of the unique species recognized in the genus *Ortegocactus*. This lineage was derived from an old ancestor (node 273, Figure 4) dated nearly 4.56 Mya (95% HPD: 2.9–6.45 Mya) in the Early Pliocene, when the cooling period ended (Figure 4). This ancestor had as its probable ancestral geographical areas the Mexican Plateau and Meridional Serranias (A and J, Figure 4), and presently this lineage is endemic to the Meridional Serranias (J, Figure 4). Lastly, the results showed that the two sampled specimens of *O. macdougallii* Alexander recently diverged about 51,000 years ago (Figure 3).

#### 3.2.7. Evolutionary Lineage 7

The ancestor of lineage 7 (node 229, Figure 4) was dated to 1.46 Mya (95% HPD: 0.6–2.48 Mya), during a time when the temperature increased slightly (ΔT~0.5 °C), and for this were estimated four probable ancestral areas (A, B, C and I, Figure 4). This lineage consists of only two northern native taxa; *M. guelzowiana* Werderm., which is endemic to the northwestern part of the continental Mexican territories; and *M. tetrancistra* Engelm. that is distributed in Baja California (B) and California (C), northwestern continental Mexican territories (I), and reaches the southern USA.

#### 3.2.8. Evolutionary Lineage 8

Lineages 8 and 9 had a common ancestor (node 271, Figure 4) that arose in Baja California 3.1 Mya (95% HPD: 1.9–4.43 Mya) (B, Figure 4). In particular, the immediate ancestor of lineage 8 (node 238, Figure 4) was dated to 1.14 Mya (0.52–1.91 Mya) during the Pleistocene, concurrently with a small increase of temperature (ΔT~1 °C) (Figure 4). Lineage 8 grouped three taxa (*M. halei* Brandegee, *M. pondii* Greene, and *M. poselgeri* Hildm.) belonging to the *Cochemiea* subgenus.

#### 3.2.9. Evolutionary Lineage 9

Lineage 9 grouped 16 taxa, all pertaining to the subgenus *Chilita* (Figure 2). Its immediate ancestor was dated to 2.77 Mya (95% HPD: 1.67–3.97 Mya) and its most probable ancestral area was Baja California (B, Figure 4). This lineage developed in the Late Pliocene when there was a slight increase in temperature (ΔT~1 °C). It diversified abundantly in Baja California. In this lineage, we identified two independent dispersal events from peninsular territories to the continental Northwest Coastal Plain (I, Figure 4). One of them occurred 1.82 Mya (node 268, Figure 4) and the other was dated to 0.01 Mya (node 239, Figure 4).

## 4. Discussion

### 4.1. Origin and Diversification of the Mammilloid Clade

The findings of this study revealed that the evolutionary history of the Mammilloid Clade (*Coryphantha*, *Escobaria*, *Mammillaria*, *Mammilloydia*, *Neolloydia*, *Ortegocactus*, and *Pelecyphora*) started ~7.5 Mya in the Miocene. During this epoch, there was a cooling trend, although the global temperature was still approximately 4–15 °C warmer than it is today [26]. The early and scarce divergence events that occurred in the Miocene were geographically restricted to the Mexican Plateau. However, during the last 4.5 million years, the cacti profusely diversified and expanded their distribution range to new areas when the global temperature was more similar to the present. Particularly, the main past colonization to new geographical areas (e.g., California, Northwest coastal plain, Pacific coast, Tehuacán Valley, and Yucatan peninsula) were dated to the last ~2.5 million years, in the Pleistocene. During this epoch various oscillations in temperature occurred [56] and have been associated with an aridity increase (e.g., [57,58]). Consequently, these cacti are modern taxa, with most of their evolutionary history occurring during the Plio-Pleistocene. In fact, the climatic oscillations in the Pleistocene were recognized as diversification driving forces for other land plants (e.g., [59,60,61,62]). Particularly for cacti, glacial [63,64] and interglacial [65,66] periods have been proposed as drivers of population processes, causing geographic contraction, isolation, and population divergence. Therefore, probably these climatic oscillations also promoted the diversification of the cacti studied here.

The Mexican Plateau has been considered geologically and climatically stable since ~15 Mya (Middle Miocene) [67]. Hence, we consider that such stability promoted the prolific speciation and colonization of cacti. However, there is a gradient of aridity along the Mexican Plateau, with the northern portion being drier than the central–southern one [68]. As cacti do not prosper in hyper-arid conditions [69], the relative “higher-humidity” at the southern end of the Mexican Plateau likely foster the ecological conditions for their abundance and speciation, which eventually led to geographic expansion. Accordingly, we postulated that the center of origin for the lineages 1 and 2 was the southern region of the Mexican Plateau, which previously was named as the Queretano-Hidalguense arid zone [70]. We based this hypothesis on the early phylogenetic split identified in these two lineages, and on their current geographical distribution. Consistent with this assumption, nearly 20% of the richness of the genus *Mammillaria* (sensu Hunt [18]) inhabit the arid lands of the Queretano-Hidalguense arid zone (Hidalgo, Guanajuato, and Querétaro) [33].

Based on similar reasoning, we inferred that the possible center of origin of lineage 3 might be the north of the Mexican Plateau. However, we recognized that more extensive taxonomic sampling is necessary to elucidate this issue. On the other hand, our results revealed that the taxa grouped in lineages 4, 7, 8 and 9 had an ecological and biogeographical affinity to northwestern Mexico. Considering that the Baja California area was the probable ancestral geographical area of lineages 8 and 9, these results suggest that this area was probably the center of origin and diversification for these lineages. Lastly, we do not discard the possibility that the small and enigmatic lineages 5 and 6 represent relicts of some phylogenetic lines that are mostly extinct. Population approaches may serve to elucidate their closest phylogenetic frontiers and recent hybridization (e.g., [17,71]). Therefore, we recommend application of this perspective to lineages 5 and 6, as well as for the sister lineages 1 and 2.

Our results also revealed that dispersal, not vicariance, was the most important past biogeographical process in these cacti. The abundant dispersal events may be related to the capacity of cacti to colonize and tolerate hostile environments (e.g., [72]), or successful seed dispersal strategies [73]. However, the data indicate that also vicariance had a relevant role in the taxa that currently are distributed in southern Mexico. Because the central portion of the Trans-Mexican Volcanic Belt (TMVB) was formed during the last three million years (plate 1 [74]), we address the TMVB as a biogeographical barrier for cacti. In fact, most of the events of colonization to southern Mexican territories were identified prior to 3 million years ago, thus the TMVB interrupted the connectivity between the arid lands of the Mexican Plateau and those of the Balsas Basin, Tehuacán Valley, and southern Meridional Serranias. In addition, the floristic affinities between the arid lands of the north and south of Mexico have been documented [53], suggesting that prior to the TMVB, the Mexican arid lands were connected from north to the south. Finally, our results showed that the arrival of cacti to the Baja California peninsula was due to dispersal and not by vicariance, as the colonization occurred later than the opening of the Gulf of California, which occurred nearly 12–6 Mya [75].

### 4.2. Taxonomic Contributions of the Phylogenetic Results

The findings of this study have explained the phylogenetic relationships of the 103 taxa, particularly the 70 taxa sampled from the genus *Mammillaria* sensu Hunt [18] were polyphyletic, as was identified previously (e.g., [35,36]). However, based on our results, the monophyly of this genus can be identified within a subset of the 70 taxa sampled as *Mammillaria*. We consider that the putative genus *Mammillaria* is represented by lineage 2, in which 85% of the taxa were from subgenus *Mammillaria*. Accordingly, monophyletic *Mammillaria* is not restricted exclusively to the *Mammillaria* subgenus, as Crozier [36] proposed, but also includes taxa of another five subgenera: *Dolichothele*, *Krainzia*, *Mammillopsis*, *Oehmea*, and *Phellosperma*. Recently, Breslin et al. [38] proposed a monophyletic circumscription of the genus *Mammillaria*, based on massive sequencing of the chloroplast genome and 52 taxa assumed to be from members of the genus. Because these 52 taxa exhibited polyphyletic relationships, these authors decided to exclude a substantial number of them in order to reach a monophyletic group.

In addition, Breslin et al. [38] proposed that the 36 taxa of the genus *Mammillaria* that were placed out of the monophyletic group, should be placed in the genus *Cochemiea* together with *N. conoidea* and *O. macdougallii*, although the species of these genera exhibit strong morphological variation (Table S1) [21]. Our results showed that lineages 4 to 9 were grouped in a distinct clade, independent of the clade that grouped lineages 1 and 2. These six lineages composed a monophyletic group (lineages 4–9). Although, these six lineages were grouped similarly to the clade named as *Cochemiea* by Breslin et al. [38] we do not agree to put together the taxa of these six lineages as our results showed strong disparities in the biogeographical history and ecologic affinities. Additionally, their strong morphological variations do not accomplish the unambiguous practical delimitation (i.e., taxonomic predictability) and stability that are required at the genus level [76]. We consider that based on a purely phylogenetic perspective, the proposal of Breslin et al. [38] to include in *Cochemiea* other taxa recognized as *Mammillaria*, *Neolloydia* and *Ortegocactus* is feasible. However, our results identified six lineages in the clade *Cochemiea* sensu Breslin et al. [38], and for us these may represent more than one genus: *Cochemiea* (lineages 7, 8 and 9), *Neolloydia* (5), *Ortegocactus* (6), and *Phellosperma* (4). These two contrasting stances exhibit the degree of subjectivity to establish the supraspecific taxonomic delimitation as has been discussed [76]. We consider that future phylogenetic studies are still necessary, and they must include specimens of the type *M. mammillaris*, and have a higher taxonomic sampling, especially of those taxa that are currently distributed in the west side of Mexican territories along the Pacific Coast. Consequently, we considered that the taxonomic circumscription of *Mammillaria* still remains unresolved. Lastly, our phylogenetic results partially supported the infrageneric classification of *Mammillaria* proposed by Hunt [18]. Accordingly, the taxa of the subgenera *Cochemiea* and *Chilita* were monophyletic. Although all the taxa of the subgenus *Mammillaria* were grouped, the monotypic and small subgenera (*Dolichothele*, *Mammillopsis* and *Oehmea*) as well as some taxa of *Phellosperma* and *Krainzia* were inserted among the species of the *Mammillaria* subgenus. In addition, because we included the raw data attached to Breslin et al. [38], we observed that in our phylogenetic tree, some of their specimens belonging to the same species were placed in discordant positions (*M. grahamii* subsp. *sheldonii* (Britton & Rose) D.R. Hunt (35158, 35161), *M. goodridgii* (35106, 35115, 35167), *M. albicans* Dawson (35107, 35103), *M. armillata* K.Brandegee (35093, 35144, 35089), *M. dioica* K.Brandegee (35170, 35131, 35119), and *M. heyderi* Muehlenpf. (16460)); this indicates the probable wrong taxonomic identification of their specimens, thus we overlooked these for the taxonomic discussion.

Recently, the study of Sánchez et al. [77] obtained a phylogenetic tree based on five chloroplast loci and eight morphologic characters. It showed that *Coryphantha* was a monophyletic genus, when excluding *C. macromeris* (Engelm.) Lem. Moreover, this last taxon and the taxa of *Escobaria* and *Pelecyphora* were grouped in the same clade, sister to *Coryphantha*, and were reclassified as a single genus (*Pelecyphora*). Our study also showed that *Coryphantha* is a monophyletic taxon, whereas *Escobaria* is paraphyletic with respect to *Coryphantha* and *Pelecyphora.* These discordances may be the result of distinct taxonomic and molecular sampling between the two studies. Nonetheless, it may be necessary to analyze morphological, ecological, and anatomical characters in order to solve these taxonomic issues.

## 5. Conclusions

We identified that the biogeographical processes, past climate conditions from the Miocene, and the recent emergence of the central portion of the TMVB strongly shaped the evolutionary history of the Mammilloid Clade (*Coryphantha*, *Escobaria*, *Mammillaria*, *Mammilloydia*, *Neolloydia*, *Ortegocactus*, and *Pelecyphora*). The past Mexican arid lands were key to providing ecological suitability for prolific cacti diversification. In these regions, they became abundant and ubiquitous elements of the arid flora. The large Mexican Plateau has been the primary evolutionary scenario for cacti, and this area is key to understand the diversity of cacti in Mexico, southern USA, Caribbean, and South America. Lastly, the Mexican territories harbor most of the world’s richness of cacti, and it is urgent to protect these arid lands, particularly the region included in the northern part of Guanajuato, Hidalgo, and Querétaro, and southern of San Luis Potosí. Our findings indicate that the genus *Mammillaria* sensu Hunt [18] is taxonomically composed of distinct evolutionary lineages, whose phylogenetic relationships require more detailed studies to reach a precise taxonomic circumscription. In this light, we consider that it is premature to undertake nomenclatural changes in *Mammillaria*, *Mammilloydia*, *Neolloydia*, and *Ortegocactus* [38,78], and such changes will bring more confusion. Therefore, we recommend maintaining the conventional taxonomic classifications (e.g., [18]) until more robust studies are undertaken. In summary, we conclude that the taxonomic circumscription of the genus *Mammillaria* still needs more work, based on phylogenetic analyses encompassed with robust and detailed ecological studies of the current geographical distribution, past niche modeling, reproductive barriers, and a clear set of diagnostic morphological characters.

## Figures and Tables

**Table 1:** Taxon diversity sampled for the seven genera. Taxonomic names and the total number of recognized taxa for the levels of genus and subgenus following Hunt [18]. Total number of the taxa and number of genomes analyzed (in silico plus those de novo sequenced); and the number of genomes de novo sequenced. NA indicates that the subgenus level is not recognized.

Genus	Subgenus	Number of Recognized Taxa	Total Number of Analyzed Taxa	Number of Genomes Analyzed	Number of Genomes de novo Sequenced
1. Coryphantha		42	10	11	3
	1.1 Coryphantha	26	8	9	2
	1.2 Neocoryphantha	15	2	2	1
2. Escobaria	NA	19	8	9	2
3. Mammillaria		163	70	105	37
	3.1. Chilita *	18	16	33	3
	3.2. Cochemiea *	3	3	10	2
	3.3 Dolichothele	6	2	2	2
	3.4. Krainzia	12	5	5	2
	3.5. Mammillaria	117	37	46	26
	3.6. Mammillopsis	1	1	1	0
	3.7. Oehmea	1	1	1	1
	3.8. Phellosperma	5	5	7	1
4. Mammilloydia	NA	1	1	1	1
5. Neolloydia *	NA	2	2	3	2
6. Ortegocactus *	NA	1	1	2	1
7. Pelecyphora	NA	2	1	1	1

* Taxa included in *Cochemiea* according to Breslin et al. [38].

## Data Availability

The raw data of the 49 samples de novo sequenced are available in the NCBI database under BioProject PRJNA934337. The SRA accessions for each sample are shown in Table S1.

## Supplementary Materials

The following supporting information can be downloaded at https://www.mdpi.com/article/10.3390/biology12040512/s1. Table S1: List and origin of the studied taxa; File S1: Geographical distribution of the 70 sampled taxa of *Mammillaria*; Table S2: List of 52 loci used for phylogenomic analysis; File S2: Output of the evaluation of six biogeographical models with BioGeoBEARS implemented in RASP4; Figure S1: Full results of ancestral geographical distribution estimated under the DEC model. Figure S2: Results of ancestral geographical distribution estimated under the DEC+J model.
