# Supplementary material for: Phylogenomics and Biogeography of the Mammilloid Clade Revealed an Intricate Evolutionary History Arose in the Mexican Plateau
Source: Biology (Basel). 2023 Mar 29;12(4):512. doi: 10.3390/biology12040512 (PMC10135466; doi:10.3390/biology12040512)
Supplement: Supplementary file 1 [file biology-12-00512-s001.zip › File S2.pdf]

File S2: Output of the evaluation of four biogeographic models with BioGeoBEARS implemented in RASP4.

#Results of Model Test#

**Model**	**LnL**	**numparams**	**d**	**e**	**j**	**AICc**	**AICc_wt**
DEC	-554.8	2	0.025	1.0e-12	0	1114	3.2e-36
DEC+J	-554.7	3	0.025	1.0e-12	0.0003	1116	1.2e-36
DIVALIKE	-604.2	2	0.030	1.0e-12	0	1213	1.0e-57
DIVALIKE+J	-603.8	3	0.029	1.0e-12	0.0011	1214	5.5e-58
