# Supplementary material for: Phylogenomics and Biogeography of the Mammilloid Clade Revealed an Intricate Evolutionary History Arose in the Mexican Plateau
Source: Biology (Basel). 2023 Mar 29;12(4):512. doi: 10.3390/biology12040512 (PMC10135466; doi:10.3390/biology12040512)
Supplement: Supplementary file 1 [file biology-12-00512-s001.zip › Table S1.pdf]

Table S1. List of the studied taxa and source of analyzed data. Names in parentheses in the third column indicate the specimen name as was originally published when it differs for the accepted name. Accession number indicates the record of the sequencing raw reads or the complete chloroplast genome sequence available at NCBI repository. Subgenus category is shown according to Hunt [12]. The last column shows representative images for the genera and subgenera level.

Genus	Subgenus	Specimen name	Taxonomic authority	Source	Accession number	Representative image
Acharagma		*A. roseanum* subsp. *galeanense* 35149	(Haugg) D.R.Hunt	Breslin et al., 2021	SRR12935149	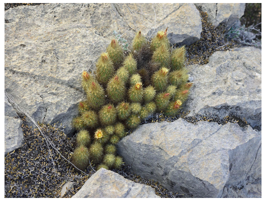<br>*A. roseanum*. By Ad Konings https://www.inaturalist.org/observations/46914915 (CC BY-NC 4.0)
		A. roseanum subsp. *galeanense* 35162	(Haugg) D.R.Hunt	Breslin et al., 2021	SRR12935162	
Ariocarpus		*A. retusus*	Scheidw.	Breslin et al., 2021	SRR12935164	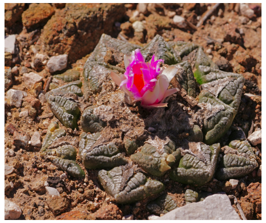<br>*A. fissuratus* By Benjamin Schwartz https://www.inaturalist.org/observations/140050488 (CC BY-NC 4.0)

Blossfeldia		*B. liliputana*	Werderm.	This study	SRR23441695	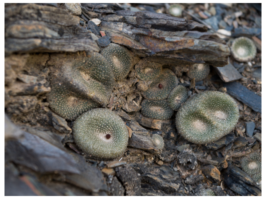 *B. liliputana*. By Martin Lowry https://www.inaturalist.org/observations/38390881 CC BY-NC 4.0)
Coryphantha	*Coryphantha*	*C. cornifera*	Lem.	Breslin et al., 2021	SRR12935099	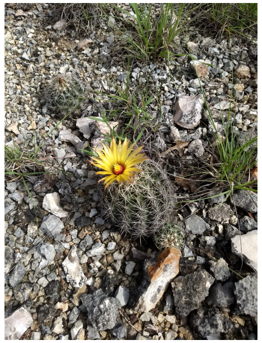 *C. sulcata*. By Ben Clement https://www.inaturalist.org/observations/81338667 (CC0 1.0)
		C. delaetiana	A.Berger	This study	SRR23441683	
		C. durangensis	(Ruenge ex Schum.) Britton & Rose	Breslin et al., 2021	SRR12935157	
		C. echinus	(Engelm.) Orcutt	Breslin et al., 2021	SRR12935176	
		C. elephantidens	Lem.	Breslin et al., 2021	SRR12935165	
		C. pallida	Britton & Rose	Breslin et al., 2021	SRR12935156	
		C. pallida 20	Britton & Rose	This study	SRR23441652	
		C. recurvata	(Engelm.) Britton & Rose	Breslin et al., 2021	SRR12935166	
		C. sulcata	Britton & Rose	Breslin et al., 2021	SRR13416461	

Neocoryphantha	*C. clavata*	(Scheidw.) Backeb.	This study	SRR23441694	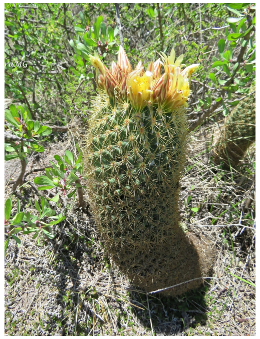 *C. erecta*. By Ma. Eugenia Mendiola González https://www.inaturalist.org/observations/52319013 (CC BY-NC-SA 4.0)
	C. erecta	(Lem. ex Pfeiff.) Lem.	Breslin et al., 2021	SRR12935151	
Cumarinia	*C. odorata*	(Boed.) Buxb.	Breslin et al., 2021	SRR12935150	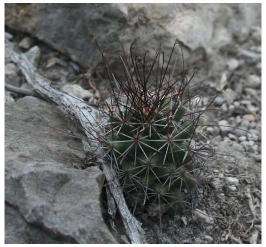 *C. odorata*. By Pedro Nájera Quezada https://www.inaturalist.org/observations/60033455 (CC BY-NC 4.0)

Escobaria	*E. chihuahuensis*	Britton & Rose	Breslin et al., 2021	SRR12935122	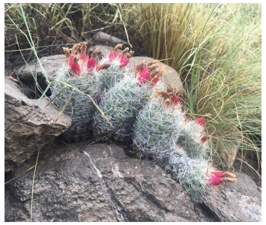 *E. tuberculosa*. By Patrick Alexander https://www.inaturalist.org/observations/33379348 (CC0 1.0)
	E. chlorantha (*Coryphantha chlorantha*)	(Engelm.) Britton & Rose	Breslin et al., 2021	SRR12935127	
	E. dasyacantha	(Engelm.) Britton & Rose	This study	SRR23441672	
	E. laredoi	(Glass & R.A.Foster) N.P.Taylor	This study	SRR23441661	
	E. robbinsorum (*Coryphantha robbinsorum*)	(W.H.Earle) A.Zimm.	Breslin et al., 2021	SRR13416462	
	E. tuberculosa	(Engelm.) Britton & Rose,	Breslin et al., 2021	SRR12935113	
	E. vivipara (*Coryphantha vivipara*)	(Nutt.) Buxb.	Breslin et al., 2021	SRR13416463	
	E. vivipara subsp. *alversonii* (*Coryphantha alversonii*)	(J.M. Coult.) D.R. Hunt	Breslin et al., 2021	SRR12935160	
	E. zilziana	(Boed.) Backeb.	Breslin et al., 2021	SRR12935125	
Lophophora	*L. williamsii*	(Lem. ex Salm-Dyck) J.M. Coult.	Breslin et al., 2021	SRR12935152	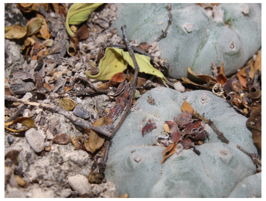 *L. williamsii*. By Pedro Nájera Quezada https://www.inaturalist.org/observations/108476028 (CC BY-NC 4.0)

Mammillaria	*Chilita*	*M. albicans* 35103	(Britton & Rose) A. Berger	Breslin et al., 2021	SRR12935103	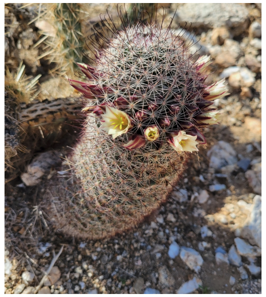<br>*M. dioica*. By Dan Horowitz https://www.inaturalist.org/observations/108563404 (CC0 1.0)
		M. albicans 35107	(Britton & Rose) A. Berger	Breslin et al., 2021	SRR12935107	
		M. armillata 35089	K. Brandegee	Breslin et al., 2021	SRR12935089	
		M. armillata 35095	K. Brandegee	Breslin et al., 2021	SRR12935095	
		M. armillata 35120	K. Brandegee	Breslin et al., 2021	SRR12935120	
		M. armillata 35144	K. Brandegee	Breslin et al., 2021	SRR12935144	
		M. blossfeldiana	Boed.	Breslin et al., 2021	SRR12935112	
		M. boolii	G.E. Linds.	Breslin et al., 2021	SRR12935148	
		M. capensis	(H.E. Gates) Craig	Breslin et al., 2021	SRR12935169	
		M. cerralboa	(Britton & Rose) Orcutt	Breslin et al., 2021	SRR12935094	
		M. dioica 35119	K. Brandegee	Breslin et al., 2021	SRR12935119	
		M. dioica 35131	K. Brandegee	Breslin et al., 2021	SRR12935131	
		M. dioica 35170	K. Brandegee	Breslin et al., 2021	SRR12935170	
		M. dioica subsp. *estebanensis* (*Mammillaria estebanensis*)	(G.E. Linds.) D.R. Hunt	Breslin et al., 2021	SRR12935168	
		M. goodridgii	Scheer	Breslin et al., 2021	SRR12935115	
		M. goodridgii var. *rectispina* 35106	Dawson	Breslin et al., 2021	SRR12935106	
		M. goodridgii var. *rectispina* 35167	Dawson	Breslin et al., 2021	SRR12935167	
		M. grahamii	Engelm.	Breslin et al., 2021	SRR12935133	
		M. grahamii 20	Engelm.	This study	SRR23441685	
		M. grahamii subsp. *sheldonii*	(Britton & Rose) D.R.Hunt	This study	SRR23441676	

	M. grahamii subsp. *sheldonii* 35158 (*Mammillaria sheldonii*)	(Britton & Rose) D.R.Hunt	Breslin et al., 2021	SRR12935158	
	M. grahamii subsp. *sheldonii* 35161 (*Mammillaria sheldonii*)	(Britton & Rose) D.R.Hunt	Breslin et al., 2021	SRR12935161	
	M. hutchisoniana 35118	(H.E. Gates) Boed.	Breslin et al., 2021	SRR12935118	
	M. hutchisoniana 35173	(H.E. Gates) Boed.	Breslin et al., 2021	SRR12935173	
	M. hutchisoniana subsp. *bullardiana* (*Mammillaria bullardiana*)	(H.E.Gates) Repp.	Breslin et al., 2021	SRR12935091	
	M. insularis	H.E. Gates	Breslin et al., 2021	SRR12935175	
	M. mainiae	K. Brandegee	Breslin et al., 2021	SRR12935121	
	M. mazatlanensis	K. Schum.	This study	SRR23441677	
	M. phitauiana 35105	(E.M. Baxter) Werderm.	Breslin et al., 2021	SRR12935105	
	M. phitauiana 35111	(E.M. Baxter) Werderm.	Breslin et al., 2021	SRR12935111	
	M. schumannii	Hildm.	Breslin et al., 2021	SRR12935090	
	M. slevinii	(Britton & Rose) Boed.	Breslin et al., 2021	SRR12935110	
	M. thornberi subsp. *yaquensis*	(R.T. Craig) D.R. Hunt	Breslin et al., 2021	SRR12935128	
Cochemiea	*M. halei*	Brandegee	Breslin et al., 2021	SRR12935093	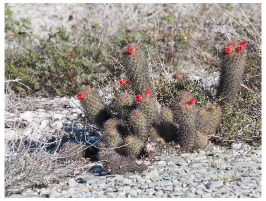 *M. halei*. By Ivan Phillipsen https://www.inatural
	M. pondii subsp. *maritima* (*Mammillaria maritima*)	(G.E.Linds.) D.R.Hunt	Breslin et al., 2021	SRR12935102	
	M. pondii 20	Greene	This study	SRR23441667	
	M. pondii 35087	Greene	Breslin et al., 2021	SRR12935087	
	M. pondii 35172	Greene	Breslin et al., 2021	SRR12935172	

	M. pondii subsp. *setispina* 35114	(J.M. Coult.) D.R. Hunt	Breslin et al., 2021	SRR12935114	ist.org/observations/ 38674775 (CC BY-NC 4.0)
	M. poselgeri 20	Hildm.	This study	SRR23441666	
	M. poselgeri 35117	Hildm.	Breslin et al., 2021	SRR12935117	
	M. poselgeri 35143	Hildm.	Breslin et al., 2021	SRR12935143	
	M. poselgeri 35145	Hildm.	Breslin et al., 2021	SRR12935145	
Dolichothele	*M. baumii*	Boed.	This study	SRR23441649	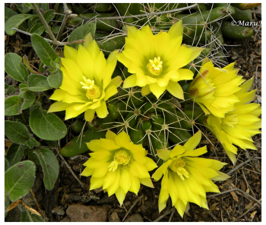 *M. longimamma*. By Ma. Eugenia Mendiola González https://www.inaturalist.org/observations/ 47207187 (CC BY-SA 4.0)
	M. longimamma	DC.	This study	SRR23441680	
Krainzia	*M. albiflora*	Backeb.	Solórzano et al., 2019	MN517610.1	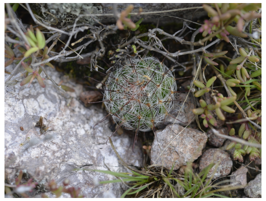 *M. longiflora*. By Ulises Guzmán https://www.inaturalist.org/observations/
	M. napina	J.A. Purpus	This study	SRR23441674	
	M. pectinifera	F.A.C. Weber	Solórzano et al., 2019	MN519716.1	
	M. solisioides	Backeb.	Solórzano et al., 2019	MN518341.1	
	M. theresae	Cutak	This study	SRR23441660	

					37046888 (CC BY-NC 4.0)
Mammillaria	*M. albicoma*	Boed.	This study	SRR23441651	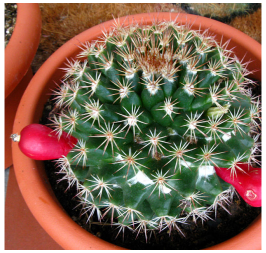 *M. mammillaris* “M. mammillaris” by Lourdes https://commons.wikimedia.org/wiki/File:Mammillaria_mammillaris.jpg (CC BY 2.0)
	M. bocasana	Poselger	This study	SRR23441647	
	M. bocensis	Craig	Breslin et al., 2021	SRR12935135	
	M. bombycina	Quehl	This study	SRR23441693	
	M. bombycina subsp. *perezdelarosae* (*Mammillaria perezdelarosae*)	(Bravo & Scheinvar) D.R.Hunt	Breslin et al., 2021	SRR12935132	
	M. brandegeei 35092	(J.M. Coult.) K. Brandegee	Breslin et al., 2021	SRR12935092	
	M. brandegeei 35098	(J.M. Coult.) K. Brandegee	Breslin et al., 2021	SRR12935098	
	M. crinita	DC.	This study	SRR23441691	
	M. columbiana subsp. yucatanensis	(Britton & Rose) D.R.Hunt	Breslin et al., 2021	SRR13416466	
	M. crucigera	Mart.	Solórzano et al., 2019	MN517613.1	
	M. decipiens	Scheidw.	This study	SRR23441690	
	M. discolor subsp. *ochoterenae*	(Bravo) U. Guzmán	This study	SRR23441671	
	M. elongata	DC.	This study	SRR23441688	
	M. evermanniana	(Britton & Rose) Orcutt	Breslin et al., 2021	SRR12935108	
	M. heyderi subsp. *gaumeri*	(Britton & Rose) D.R.Hunt	This study	SRR23441687	
	M. heyderi 16460	Muehlenpf.	Breslin et al., 2021	SRR13416460	
	M. heyderi subsp. *heyderi* 20	Muehlenpf.	This study	SRR23441684	
	M. huitzilopochtli	D.R. Hunt	Breslin et al., 2021	SRR12935159	
	M. huitzilopochtli 17	D.R. Hunt	Solórzano et al., 2019	MN517612.1	

	M. huitzilopochtli 20	D.R. Hunt	This study	SRR23441682
	M. johnstonii 35129	(Britton & Rose) Orcutt	Breslin et al., 2021	SRR12935129
	M. johnstonii 35130	(Britton & Rose) Orcutt	Breslin et al., 2021	SRR12935130
	M. lasiacantha	Engelm.	This study	SRR23441681
	M. magnifica	F.G. Buchenau	This study	SRR23441679
	M. magnimamma	Haw.	This study	SRR23441678
	M. nana	Backeb.	This study	SRR23441675
	M. nunezii	(Britton & Rose) Orcutt	This study	SRR23441673
	M. peninsularis	(Britton & Rose) Orcutt	Breslin et al., 2021	SRR12935101
	M. perbella	Hildm. ex K. Schum.	This study	SRR23441670
	M. petrophila	K. Brandegee	Breslin et al., 2021	SRR12935096
	M. petrophila subsp. *arida*	(Rose ex Quehl) D.R.Hunt	Breslin et al., 2021	SRR12935142
	M. picta	Meinsh.	This study	SRR23441669
	M. polyedra	Mart.	This study	SRR23441668
	M. pottsii	Scheer ex Salm-Dyck	Breslin et al., 2021	SRR12935141
	M. prolifera	(Mill.) Haw.	Breslin et al., 2021	SRR13416465
	M. sartorii	J.A.Purpus	This study	SRR23441665
	M. schiedeana	Ehrenb. ex Schltdl	This study	SRR23441664
	M. scrippsiana	(Britton & Rose) Orcutt	This study	SRR23441663
	M. sphacelata	Mart.	Breslin et al., 2021	SRR12935136
	M. sphacelata 20	Mart.	This study	SRR23441662

	M. supertexta	Mart. ex Pfeiff	Solórzano et al., 2019	MN508963.1	
	M. tayloriorum	Glass & R.A. Foster	Breslin et al., 2021	SRR12935097	
	M. uncinata	Zucc. ex Pfeiff.	This study	SRR23441653	
	M. vetula subsp. *gracilis*	(Pfeiff.) D.R.Hunt	This study	SRR23441686	
	M. voburnensis subsp. *eichlamii*	Quehl	This study	SRR23441689	
	M. wiesingeri	Boed.	This study	SRR23441659	
Mammillopsis	*M. senilis*	Lodd. ex Salm-Dyck	Breslin et al., 2021	SRR12935123	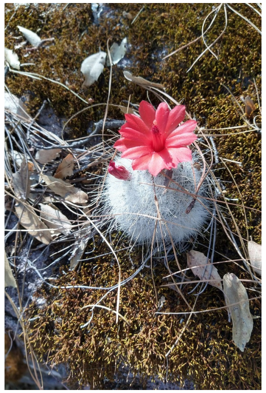 *M. senilis*. By Selene Jazmin Delgado Corral https://www.inaturalist.org/observations/131919916 (CC BY-NC 4.0)

Oehmea	*M. beneckei*	Ehrenb.	This study	SRR23441648	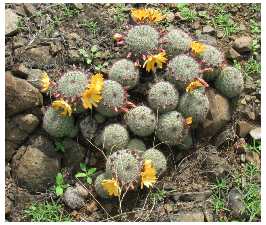 *M. beneckei*. By Caja de Zicuirán https://www.inaturalist.org/observations/128801487 (CC BY-NC 4.0)
Phellosperma	*M. barbata*	Engelm.	This study	SRR23441650	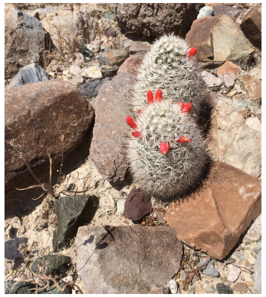 *M. tetrancistra*. By Andy Kleinhesselink https://www.inaturalist.org/observations/5649546 (CC0 1.0)
	M. barbata var. *viridiflora* (*Mammillaria viridiflora*)	Engelm.	Breslin et al., 2021	SRR12935155	
	M. guelzowiana	Werderm.	Breslin et al., 2021	SRR12935138	
	M. tetrancistra 35140	Engelm.	Breslin et al., 2021	SRR12935140	
	M. wrightii	Engelm.	Breslin et al., 2021	SRR12935147	
	M. wrightii subsp. *wilcoxii*	(Toumey ex K. Schum.) D.R. Hunt	Breslin et al., 2021	SRR12935134	
	M. zephyranthoides	Scheidw.	Solórzano et al., 2019	MN517611.1	

Mammilloydia	*M. candida*	(Scheidw.) Buxb.	This study	SRR23441692	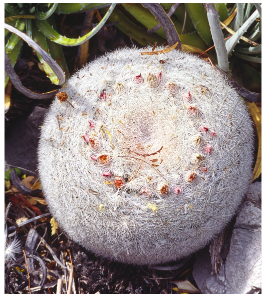 *M. candida*. By Alessandro Mosco https://www.inaturalist.org/observations/126613439 (CC BY-NC 4.0)
Neolloydia	*N. conoidea*	(DC.) Britton & Rose	Breslin et al., 2021	SRR12935153	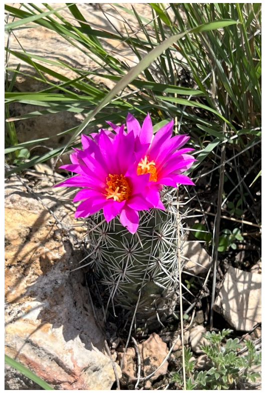 *N. conoidea*. By CK Kelly https://www.inaturalist.org/observations/133038218 (CC BY 4.0)
	N. conoidea 20	(DC.) Britton & Rose	This study	SRR23441658	
	N. matehualensis	Backeb.	This study	SRR23441657	

Ortegocactus	*O. macdougallii*	Alexander	Breslin et al., 2021	SRR12935137	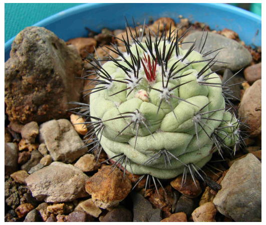<br>*O. macdougallii.* "Ortegocactus macdougalli" by Guillermo Huerta Ramos https://www.flickr.com/photos/xerofilo/353430608/in/photolist-Gy5QqQ-UDjnVp-xeqBb-yik2b-Djo7Zq-wAUaio (CC BY-NC 2.0)
	O. macdougallii 20	Alexander	This study	SRR23441656	

Pelecyphora	*P. strobiliformis*	(Werderm.) Frič & Schelle ex Kreuz.	This study	SRR23441655	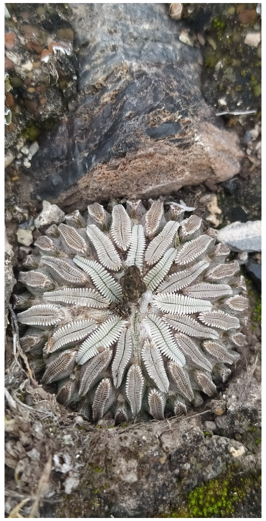 *P. aselliformis*. By paola_pineda https://www.inaturalist.org/observations/121156391 (CC BY-NC 4.0)
Stenocactus	*S. multicostatus* subsp. *zacatecasensis*	(Britton & Rose) U.Guzmán & Vázq.	This study	SRR23441654	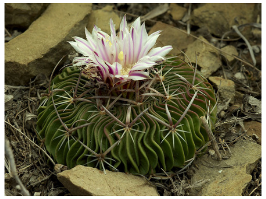 *S. multicostatus*. By Ad Konings https://www.inaturalist.org/photos/85150525

					(CC BY-NC 4.0)
Strombocactus	*S. disciformis*	(DC.) Britton & Rose	Breslin et al., 2021	SRR12935139	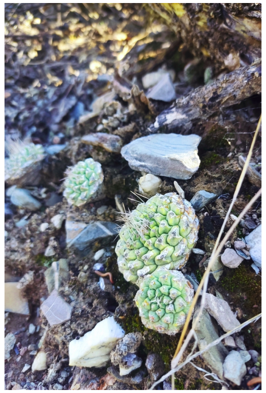 *S. disciformis.* By Jose Antonio Aranda Pineda https://www.inaturalist.org/observations/131295608 (CC BY-NC-SA 4.0)
Turbinicarpus	*T. alonsoi*	Glass & S. Arias	Breslin et al., 2021	SRR12935124	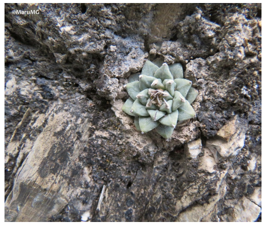 *T. alonsoi.* By Ma. Eugenia Mendiola González https://www.inatural
	T. viereckii	(Werderm.) V.John & Riha	Breslin et al., 2021	SRR12935163	

		ist.org/observations/<br>140856491<br>(CC BY-SA 4.0)
