# Supplementary material for: Phylogenomics and Biogeography of the Mammilloid Clade Revealed an Intricate Evolutionary History Arose in the Mexican Plateau
Source: Biology (Basel). 2023 Mar 29;12(4):512. doi: 10.3390/biology12040512 (PMC10135466; doi:10.3390/biology12040512)
Supplement: Supplementary file 1 [file biology-12-00512-s001.zip › Table S2.pdf]

Table S2. List of 52 loci used for phylogenomic analysis. From the alignment of each locus was calculated the length, informative sites (number and percentage) and counted bases between the studied taxa.

Locus	Length (bp)	Informative sites (bp)	Counted bases (bp)	Informative sites (%)
1. accD	5177	2072	4271	48.51
2. atpB-rbcL	2050	398	1455	27.35
3. atpE	413	73	411	17.76
4. atpF-atpH	464	81	445	18.20
5. atpH-atpI	797	123	745	16.51
6. infA	270	94	264	35.61
7. ndhF-trnN	497	94	480	19.58
8. petA-cemA	287	45	280	16.07
9. petB-petD	1114	194	1079	17.98
10. psaA-ycf3	1446	237	1109	21.37
11. psbA-trnH	488	82	324	25.31
12. psbE-petL	1533	185	1014	18.24
13. psbH-petB	1014	165	998	16.53
14. psbK-psbI	528	102	514	19.84
15. rbcL-petA	1089	160	1036	15.44
16. rpl14	369	56	369	15.18
17. rpl16-rps3	1484	320	1400	22.86
18. rpl16	453	86	453	18.98
19. rpl20	415	103	408	25.25
20. rpl22	616	164	554	29.60
21. rpl32	321	71	320	22.19
22. rpoA	1080	170	1068	15.92
23. rps11	432	134	426	31.46
24. rps14	303	51	303	16.83
25. rps15	267	72	267	26.97
26. rps18	300	124	296	41.89

27. rps19	462	102	450	22.67
28. rps2-rpoC2	301	56	295	18.98
29. rps2	753	149	742	20.08
30. rps3	753	191	741	25.78
31. rps4	651	116	636	18.24
32. rps7	504	121	480	25.21
33. rps8	432	65	417	15.59
34. trnC-petN	1616	107	642	16.67
35. trnF-psbJ	1121	172	1104	15.58
36. trnI-ycf68	1418	244	1223	19.95
37. trnI-rrn16	321	51	305	16.72
38. trnK-psbA	265	76	257	29.57
39. trnL-ycf1	300	99	294	33.67
40. trnL-trnL	732	167	678	24.63
41. trnL-rpl32	1905	467	1722	27.12
42. trnN-trnR	1349	207	1297	15.96
43. trnQ-psbK	648	71	384	18.49
44. trnS-clpP	333	60	316	18.99
45. trnS-rps4	422	85	401	21.20
46. trnS-psbZ	417	80	412	19.42
47. trnT-psbD	1473	148	783	18.90
48. trnT-trnL	917	103	584	17.64
49. trnV-rps12	1571	136	882	15.42
50. ycf1-rps15	277	58	262	22.14
51. ycf1	7913	2328	6764	34.42
52. ycf4-psaI	529	54	333	16.22
