# Supplementary figures and images for: Phylogenomics and Biogeography of the Mammilloid Clade Revealed an Intricate Evolutionary History Arose in the Mexican Plateau
Source: Biology (Basel). 2023 Mar 29;12(4):512. doi: 10.3390/biology12040512 (PMC10135466; doi:10.3390/biology12040512)

**File S1.** Geographic distribution of the 70 sampled taxa of *Mammillaria*.

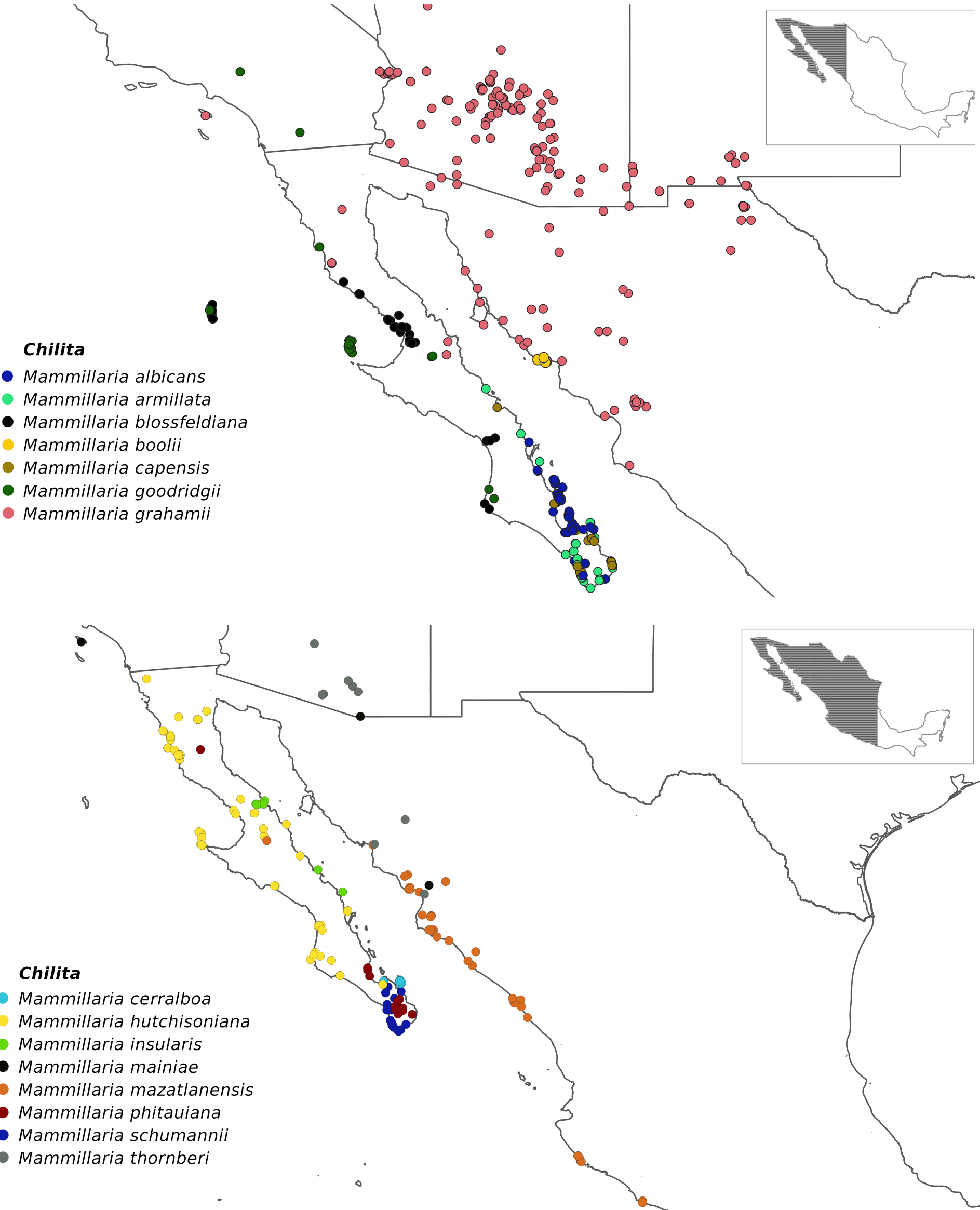

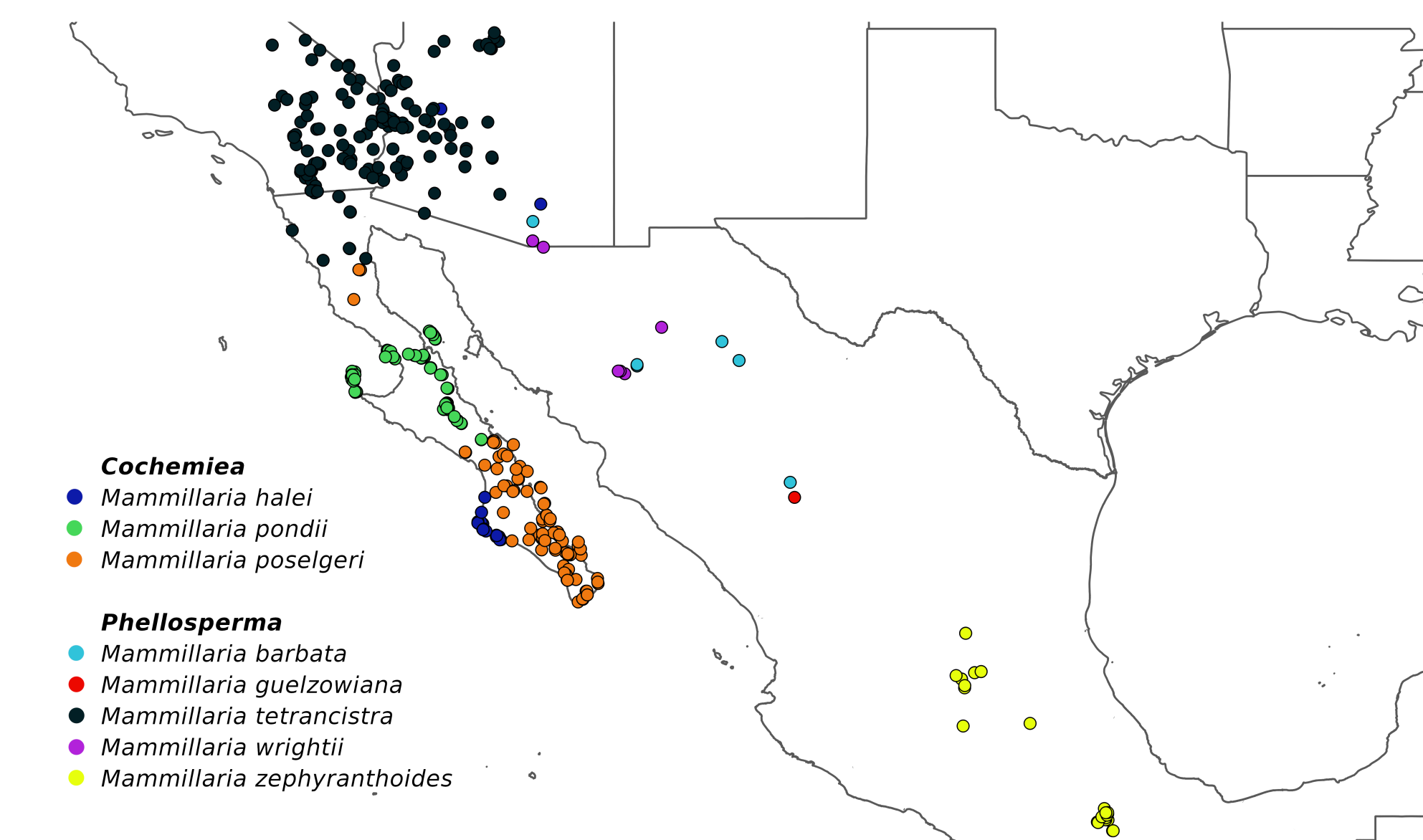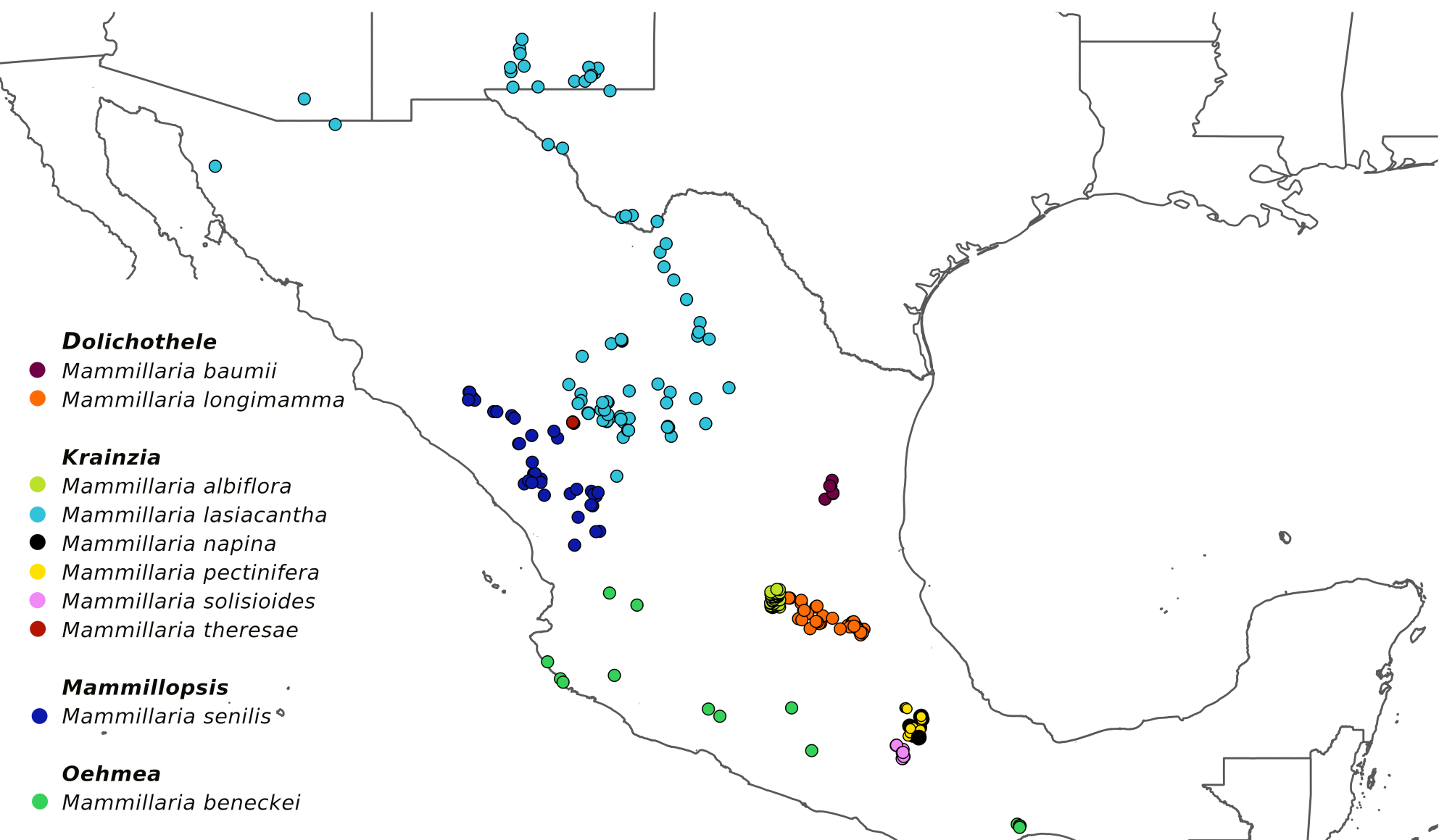

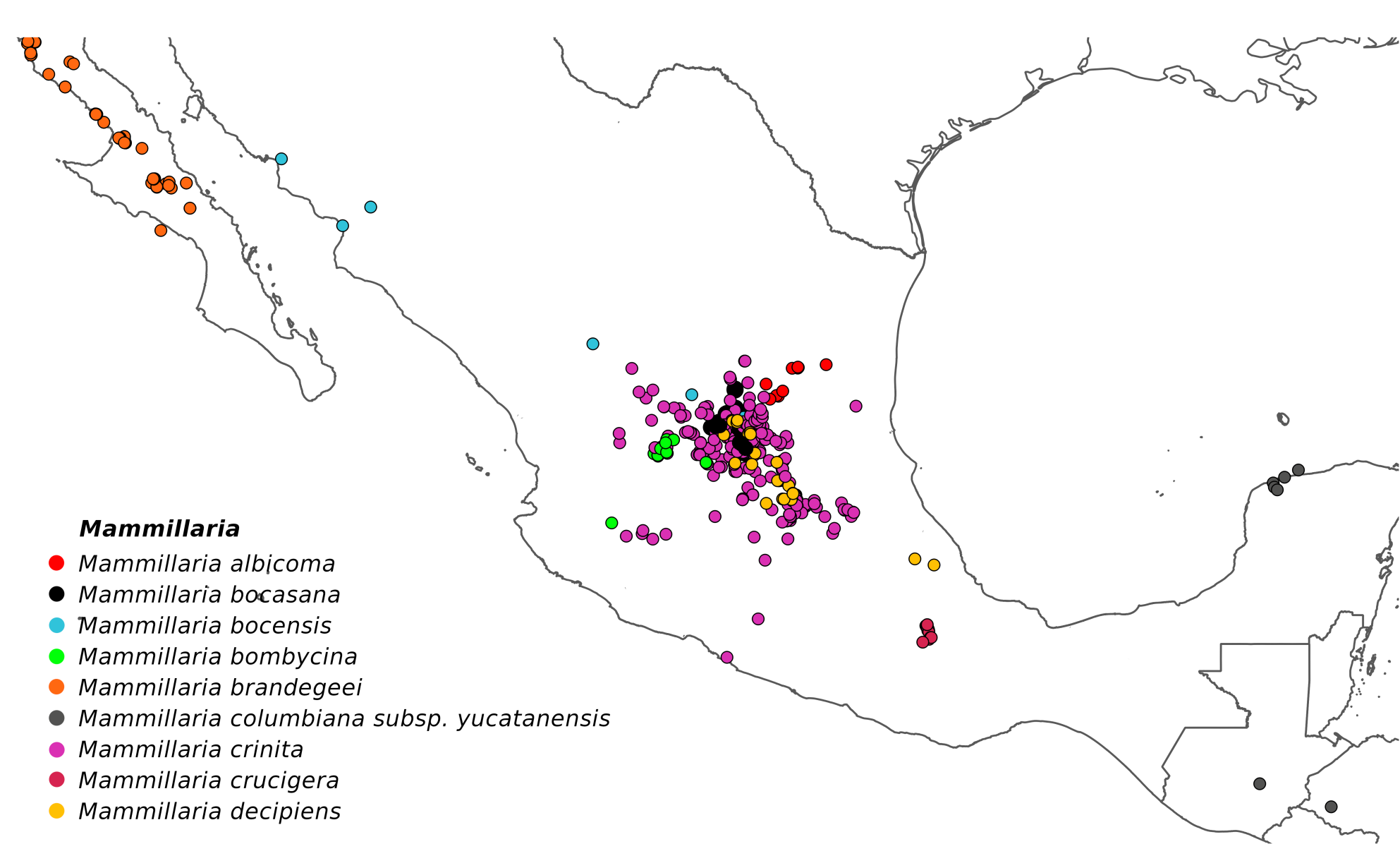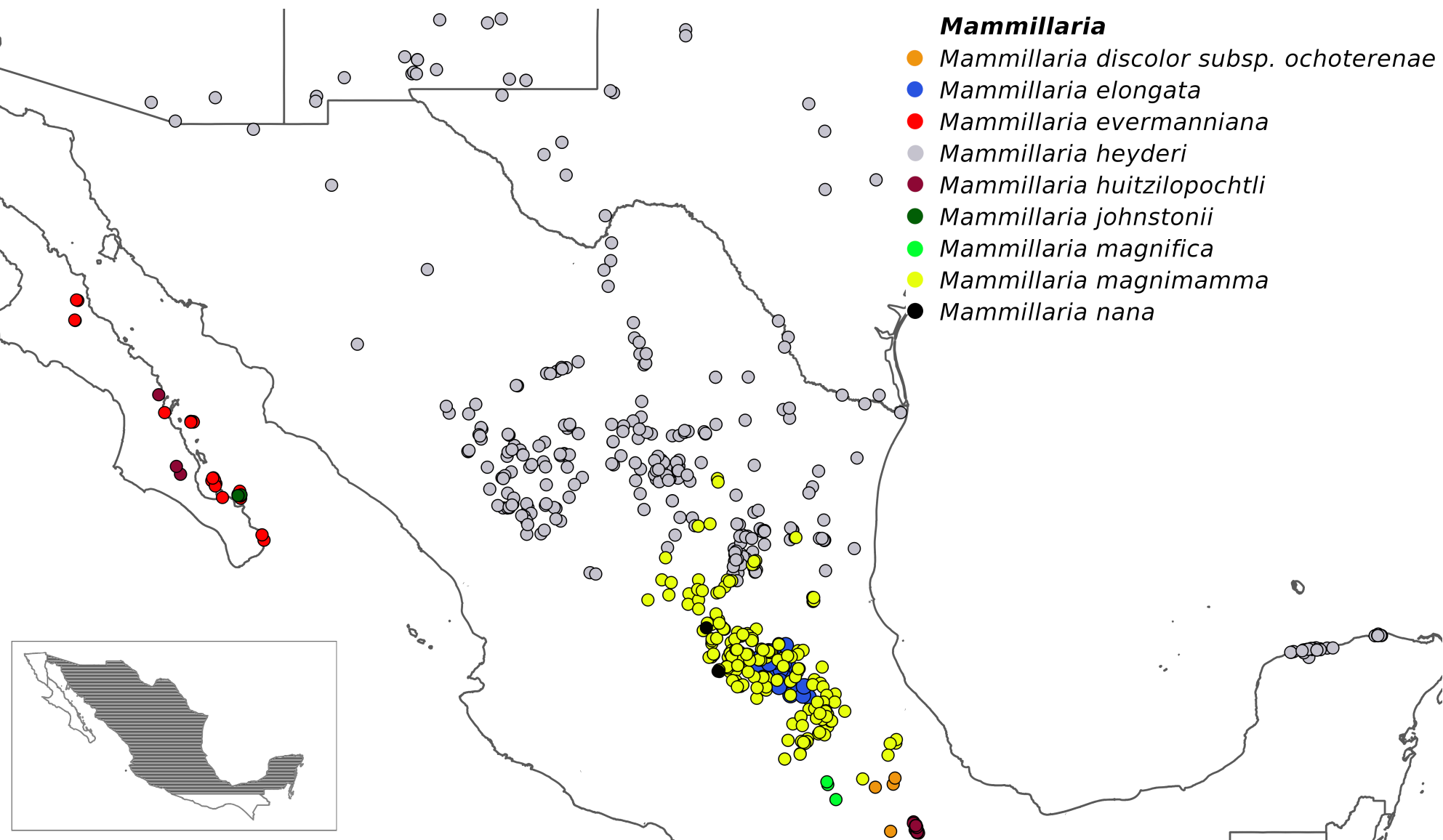

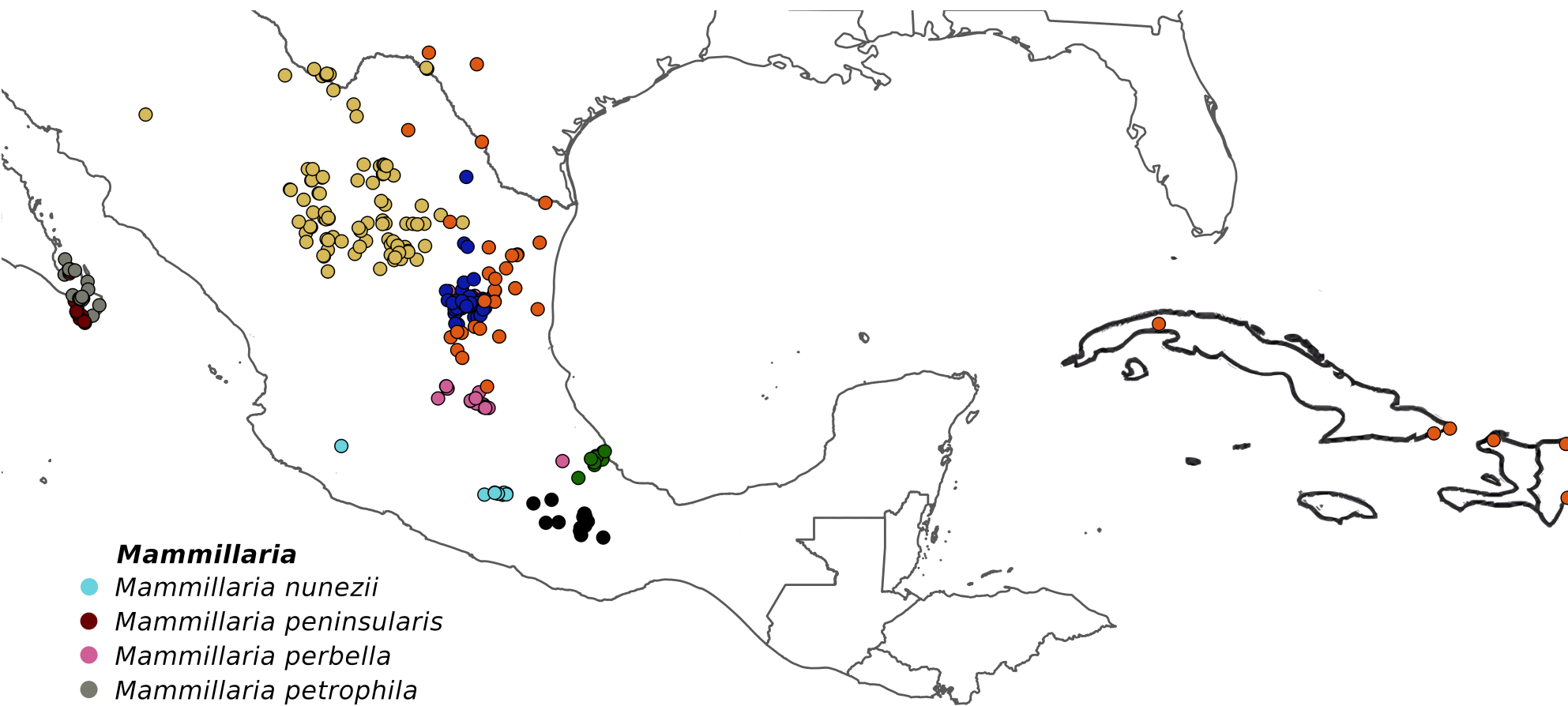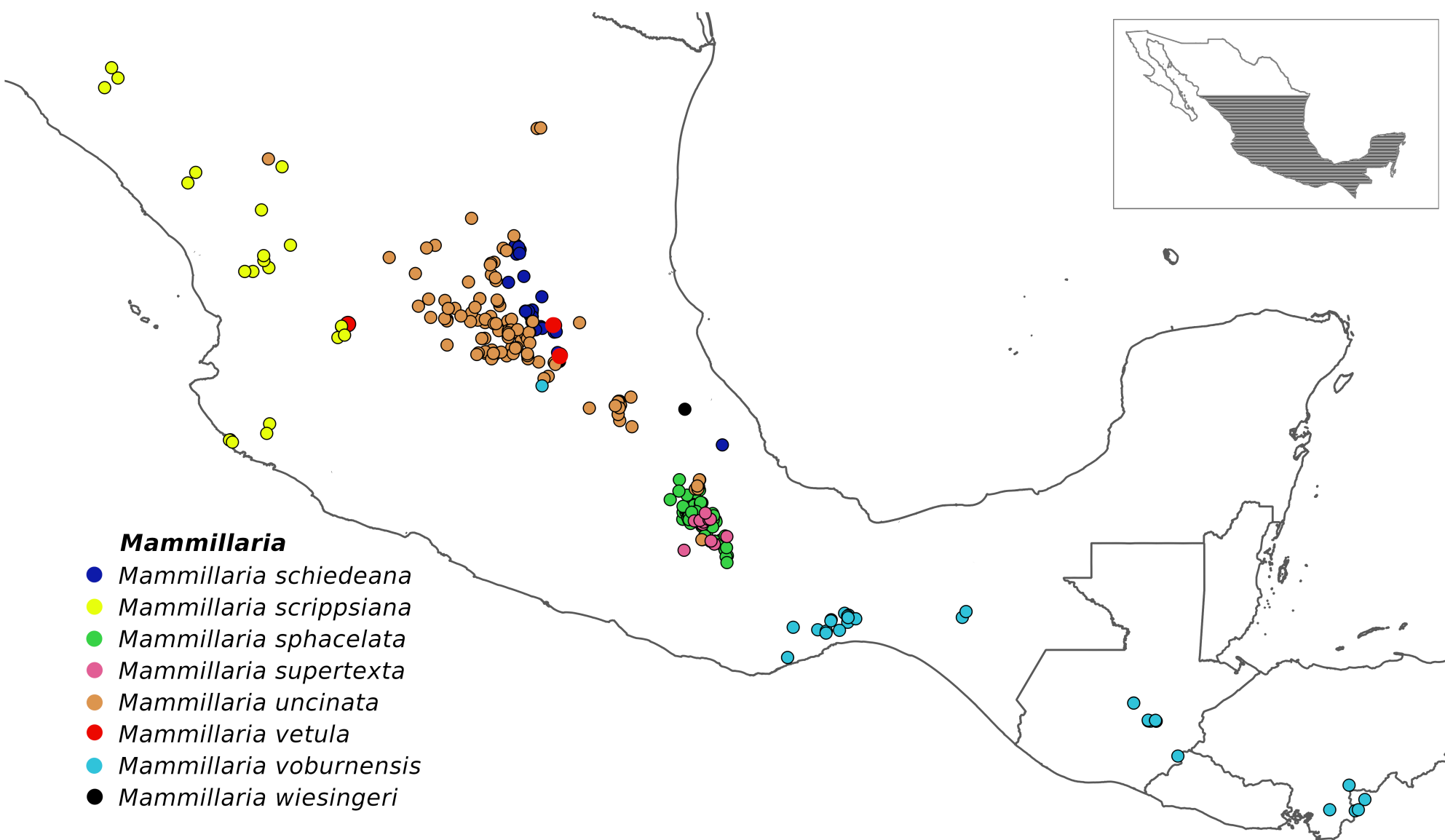

Supplement: Supplementary file 1 [file biology-12-00512-s001.zip › File S1.pdf]
